# Supplementary material for: Ultrastiff metamaterials generated through a multilayer strategy and topology optimization
Source: Nat Commun. 2024 Apr 6;15:2984. doi: 10.1038/s41467-024-47089-8 (PMC10998847; doi:10.1038/s41467-024-47089-8)
Supplement: Supplementary file 1 — Supplementary information [file 41467_2024_47089_MOESM1_ESM.docx]

**Supplementary Information for**

**Ultrastiff metamaterials generated through a multilayer strategy and topology optimization**

**Authors:**

Yang Liu (刘洋) ^1, 2^, Yongzhen Wang ^1^, Hongyuan Ren ^1^, Zhiqiang Meng ^1, 2^, Xueqian Chen ^1^, Zuyu Li ^3, 4,^ *,

Liwei Wang ^5,^ *, Wei Chen ^5,^ *, Yifan Wang ^2,^ *, and Jianbin Du ^1,^ *

**Author Affiliations:**

^1^ School of Aerospace Engineering, Tsinghua University, Beijing, PR China

^2^ School of Mechanical & Aerospace Engineering, Nanyang Technological University, Singapore

^3^ School of Automation, Guangdong University of Petrochemical Technology, Maoming, China

^4^ School of Mechanical and Mechatronic Engineering, University of Technology Sydney, Ultimo, Australia

^5^ Department of Mechanical Engineering, Northwestern University, Evanston, U.S.A

***Corresponding email:**

Zuyu Li, Email: [lizuyu@gdupt.edu.cn](mailto:lizuyu@gdupt.edu.cn)

Liwei Wang, Email: [liwei.wang@northwestern.edu](mailto:liwei.wang@northwestern.edu)

Wei Chen, Email: [weichen@northwestern.edu](mailto:weichen@northwestern.edu)

Yifan Wang, Email: [yifan.wang@ntu.edu.sg](mailto:yifan.wang@ntu.edu.sg)

Jianbin Du, Email: [dujb@tsinghua.edu.cn](mailto:dujb@tsinghua.edu.cn)

**This Supplementary Information includes:**

Supplementary Note 1: Material characterization

Supplementary Note 2: Optimization algorithm

Supplementary Note 3: Practical implementation

Supplementary Note 4: Method verification

Supplementary Note 5: Optimization anomaly

Supplementary Note 6: Disparity between experiment and simulation

**Supplementary Note 1: Material characterization**

**1.1 Effective elastic properties**

For anisotropic materials, the linear elastic relationship based on Hooke’s law ^[1]^ can be given as follows,

| $\sigma=c\cdot\varepsilon$ | (1) |
| --- | --- |

where $\sigma$ is the stress tensor, $\varepsilon$ the strain tensor, and $c$ the elastic stiffness tensor. By using the orthonormal coordinate system, Eq. is rewritten as,

| $\sigma_{ij}=c_{ijkl}\varepsilon_{kl}$ | (2) |
| --- | --- |

As the stress and strain tensors are symmetric and the stress-strain relation in linear elasticity can be derived from a strain energy density function, we have the following symmetry relationships,

| $c_{ijkl}=c_{jikl} , c_{ijkl}=c_{jilk} , c_{ijkl}=c_{klij}$ | (3) |
| --- | --- |

Based on the above relationships, the stress-strain relation for linear elastic materials can be expressed in matrix form,

| $\left[ \begin{matrix} \begin{matrix} \sigma_{11} \\ \sigma_{22} \\ \sigma_{33} \end{matrix} \\ \begin{matrix} \sigma_{23} \\ \sigma_{31} \\ \sigma_{12} \end{matrix} \end{matrix} \right]=\left[ \begin{matrix} \begin{matrix} c_{1111} & c_{1122} & c_{1133} \\ c_{2211} & c_{2222} & c_{2233} \\ c_{3311} & c_{3322} & c_{3333} \end{matrix} & \begin{matrix} c_{1123} & c_{1131} & c_{1112} \\ c_{2223} & c_{2231} & c_{2212} \\ c_{3323} & c_{3331} & c_{3312} \end{matrix} \\ \begin{matrix} c_{2311} & c_{2322} & c_{2333} \\ c_{3111} & c_{3122} & c_{3133} \\ c_{1211} & c_{1222} & c_{1233} \end{matrix} & \begin{matrix} c_{2323} & c_{2331} & c_{2312} \\ c_{3123} & c_{3131} & c_{3112} \\ c_{1223} & c_{1231} & c_{1212} \end{matrix} \end{matrix} \right]\left[ \begin{matrix} \begin{matrix} \varepsilon_{11} \\ \varepsilon_{22} \\ \varepsilon_{33} \end{matrix} \\ \begin{matrix} {2\varepsilon}_{23} \\ {2\varepsilon}_{31} \\ 2\varepsilon_{12} \end{matrix} \end{matrix} \right]$ | (4) |
| --- | --- |

In Voigt notation ^[2]^, Eq.(4) is given as,

| $\left[ \begin{matrix} \begin{matrix} \sigma_{1} \\ \sigma_{2} \\ \sigma_{3} \end{matrix} \\ \begin{matrix} \sigma_{4} \\ \sigma_{5} \\ \sigma_{6} \end{matrix} \end{matrix} \right]=\left[ \begin{matrix} \begin{matrix} C_{11} & C_{21} & C_{13} \\ C_{12} & C_{22} & C_{23} \\ C_{13} & C_{23} & C_{33} \end{matrix} & \begin{matrix} C_{14} & C_{15} & C_{16} \\ C_{24} & C_{25} & C_{26} \\ C_{34} & C_{35} & C_{36} \end{matrix} \\ \begin{matrix} C_{14} & C_{24} & C_{34} \\ C_{15} & C_{25} & C_{35} \\ C_{16} & C_{26} & C_{36} \end{matrix} & \begin{matrix} C_{44} & C_{45} & C_{46} \\ C_{45} & C_{55} & C_{56} \\ C_{46} & C_{56} & C_{66} \end{matrix} \end{matrix} \right]\left[ \begin{matrix} \begin{matrix} \varepsilon_{1} \\ \varepsilon_{2} \\ \varepsilon_{3} \end{matrix} \\ \begin{matrix} \varepsilon_{4} \\ \varepsilon_{5} \\ \varepsilon_{6} \end{matrix} \end{matrix} \right]$ | (5) |
| --- | --- |

As an orthotropic elastic material has three orthogonal symmetry planes ^[2]^, the following conditions are satisfied,

| $C_{14}=C_{15}=C_{24}=C_{25}=C_{34}=C_{35}=C_{46}=C_{56}=0$ | (6) |
| --- | --- |
| $C_{16}=C_{26}=C_{36}=C_{45}=0$ | (7) |

Then the following stiffness matrix for orthotropic elastic materials can be obtained,

| $C=\left[ \begin{matrix} \begin{matrix} C_{11} & C_{12} & C_{13} \\ C_{12} & C_{22} & C_{23} \\ C_{13} & C_{23} & C_{33} \end{matrix} & \begin{matrix} 0 & 0 & 0 \\ 0 & 0 & 0 \\ 0 & 0 & 0 \end{matrix} \\ \begin{matrix} 0 & 0 & 0 \\ 0 & 0 & 0 \\ 0 & 0 & 0 \end{matrix} & \begin{matrix} C_{44} & 0 & 0 \\ 0 & C_{55} & 0 \\ 0 & 0 & C_{66} \end{matrix} \end{matrix} \right]$ | (8) |
| --- | --- |

Cubic symmetry materials are special orthotropic materials that are invariant concerning 90° rotations with respect to the principal axes, i.e., the material is the same along its principal axes. Thus, for a cubic symmetric lattice cell, we have the following conditions,

| $C_{11}=C_{22}=C_{33} , C_{12}=C_{13}=C_{23} , C_{44}=C_{55}=C_{66}$ | (9) |
| --- | --- |

With only three independent entries, the effective elastic tensor $C$ can be rewritten as,

| $C=\left[ \begin{matrix} \begin{matrix} C_{11} & C_{12} & C_{12} \\ C_{12} & C_{11} & C_{12} \\ C_{12} & C_{12} & C_{11} \end{matrix} & \begin{matrix} 0 & 0 & 0 \\ 0 & 0 & 0 \\ 0 & 0 & 0 \end{matrix} \\ \begin{matrix} 0 & 0 & 0 \\ 0 & 0 & 0 \\ 0 & 0 & 0 \end{matrix} & \begin{matrix} C_{44} & 0 & 0 \\ 0 & C_{44} & 0 \\ 0 & 0 & C_{44} \end{matrix} \end{matrix} \right]$ | (10) |
| --- | --- |

The inverse of this stiffness matrix can be written as follows ^[3]^,

| $S=\left[ \begin{matrix} \begin{matrix} \frac{1}{E} & -\frac{\upsilon}{E} & -\frac{\upsilon}{E} \\ -\frac{\upsilon}{E} & \frac{1}{E} & -\frac{\upsilon}{E} \\ -\frac{\upsilon}{E} & -\frac{\upsilon}{E} & \frac{1}{E} \end{matrix} & \begin{matrix} 0 & 0 & 0 \\ 0 & 0 & 0 \\ 0 & 0 & 0 \end{matrix} \\ \begin{matrix} 0 & 0 & 0 \\ 0 & 0 & 0 \\ 0 & 0 & 0 \end{matrix} & \begin{matrix} \frac{1}{G} & 0 & 0 \\ 0 & \frac{1}{G} & 0 \\ 0 & 0 & \frac{1}{G} \end{matrix} \end{matrix} \right]$ | (11) |
| --- | --- |

where $E$ is the Young’s modulus and shear modulus, $\upsilon$ the Poisson’s ratio, and $G$ the shear modulus, which can be calculated by,

| $E=\frac{1}{S_{11}}$ | (12) |
| --- | --- |
| $\upsilon=-\frac{S_{12}}{S_{11}}$ | (13) |
| $G=\frac{1}{S_{44}}$ | (14) |

As illustrated in **Supplementary Fig. 1**, the effective Young's modulus and shear modulus in an arbitrary direction are given by,

| $E_{\theta\varphi}=S_{11}-2\left( S_{11}-S_{12}-\frac{1}{2}S_{44} \right)\left( l_{1}^{2}l_{2}^{2}+l_{1}^{2}l_{3}^{2}+l_{2}^{2}l_{3}^{2} \right)$ | (15) |
| --- | --- |
| $G_{\theta\varphi\chi}=4S_{11}\left( l_{1}^{2}m_{1}^{2}+l_{1}^{2}m_{2}^{2}+l_{2}^{2}m_{3}^{2} \right)+8S_{12}\left( l_{1}l_{2}m_{1}m_{2}+l_{1}l_{3}m_{1}m_{3}+l_{2}l_{3}m_{2}m_{3} \right)+S_{44}\left[ \left( l_{1}m_{2}+l_{2}m_{1} \right)^{2}+\left( l_{1}m_{3}+l_{3}m_{1} \right)^{2}+\left( l_{2}m_{3}+l_{3}m_{2} \right)^{2} \right]$ | (16) |

where,

| $l=\left( \begin{aligned} \sin\theta\cos\varphi\\ \sin\theta\cos\theta\\ \cos\theta\end{aligned} \right); m=\left( \begin{aligned} \cos\theta\cos\varphi\cos\chi-\sin\varphi\sin\chi\\ \cos\theta\sin\varphi\cos\chi+\cos\varphi\sin\chi\\ -\sin\theta\cos\chi\end{aligned} \right)$ | (17) |
| --- | --- |

**1.2 Zener ratio**

The Zener ratio has been well-accepted as an index to quantify the anisotropy property of a cubic material, using shear elastic coefficients ^[4]^. For a cubic symmetric cellular material, the Zener ratio is given as follows,

| $Z=\frac{2G\left( 1+\upsilon\right)}{E}=\frac{2C_{44}}{C_{11}-C_{12}}$ | (18) |
| --- | --- |

**1.3 Voigt upper bound**

The Voigt upper bounds ($VU$) represent the theoretical limits of the effective Young’s modulus and yield strength for anisotropic cellular materials, which are given as follows,

| $E^{VU}=E_{S}\bar{\rho}$ | (19) |
| --- | --- |
| $\sigma_{y}^{VU}=\sigma_{ys}\bar{\rho}$ | (20) |

where $\bar{\rho}$ is the relative density, $E_{S}$, $\sigma_{ys}$, the Young’s modulus, and yield strength of the constituent material, respectively.

**1.4 Hashin-Shtrikman upper bound**

The Hashin-Shtrikman upper bound ($HSU$) for the effective Young’s modulus is the theoretical limit for isotropic cellular materials ^[5-7]^. For a cubic symmetric lattice material, the $E^{HSU}$ is given as follows,

| $E^{HSU}=\frac{2E_{S}\bar{\rho}(7-5v_{s})}{15\left( \bar{\rho}-1 \right)v_{s}^{2}+2\left( \bar{\rho}-6 \right)v_{s}-13\bar{\rho}+27}$ | (21) |
| --- | --- |

**1.5 Suquet upper bound**

The Suquet upper bound ($SU$) for the effective yield strength is the theoretical limit for isotropic cellular materials ^[8]^. For a cubic symmetric lattice material, the $\sigma_{y}^{SU}$ is given as follows,

| $\sigma_{y}^{SU}=\frac{6\sigma_{ys}\bar{\rho}}{\sqrt{69-33\bar{\rho}}}$ | (22) |
| --- | --- |

**1.6 Gibson-Ashby scaling power-law fit**

The Gibson-Ashby scaling power-law fit is used to quantify the deformation mode of cellular materials ^[9]^, which is stated as,

| $E/{E_{S}}=C\bar{\rho}^{n}$ | (23) |
| --- | --- |

where $C$ is the scaling coefficient, and $n$ is the scaling exponent. For the stretching-dominated lattice, $n$ equals 1. While for bending-dominated lattice, $n$ equals 2.

**Supplementary Note 2: Optimization algorithm**

**2.1 ODE-driven level-set density method**

Regarding the optimization algorithm, a novel ordinary differential equation (ODE) driven level-set density method was employed to drive optimization ^[10]^. Due to the powerful topological variation ability, this method usually gives birth to better optimization results. The method is a combination of the level-set and density method. The level-set function (LSF) $\phi$ is used to describe the structural interface by extracting the zero level-set from the level-set function ^[12, 13]^, which is parameterized as follows,

| $\left\{ \begin{matrix} \phi\left( \mathbf{X} \right)>0 & \forall\mathbf{X}\in\Omega\backslash\partial\Omega\\ \phi\left( \mathbf{X} \right)=0 & \forall\mathbf{X}\in\partial\Omega\\ \phi\left( \mathbf{X} \right)<0 & \forall\mathbf{X}\in D\backslash\Omega\end{matrix} \right.$ | (24) |
| --- | --- |

where $\mathbf{X}$ is the coordinate of a point inside the design domain $D$ and $\partial\Omega$ is the boundary of the solid domain $\Omega$. The density field is used to develop the physical interpolation model, which is shifted from the level-set function. For the proposed method, an exact Heaviside function $H\left( \phi\right)$ is used to map the level-set function onto a physical interpolation model,

| $H\left( \phi\right)=\left\{ \begin{matrix} 1 & \mathrm{if}\phi\geq0 \\ 0 & \mathrm{if}\phi<0 \end{matrix} \right.$ | (25) |
| --- | --- |

The ersatz material model ^[12, 13]^ is adopted here for FEA for convenience as the design domain can be meshed once and for all, and involvements of mesh regeneration or modification are excluded. The transformation of the ersatz material model $\rho_{e}$ from the level-set function is given as:

| $\rho_{e}=\frac{\int_{D_{e}}H\left( \phi\right)d\Omega}{\int_{D_{e}}d\Omega}$ | (26) |
| --- | --- |

where $D_{e}$ is the element domain. We use a power law penalization scheme ^[11]^ to interpolate the element stiffness $E_{e}$,

| $E_{e}\left( \rho_{e} \right)=E_{min}+\left( \rho_{e} \right)^{p}\left( E_{0}-E_{min} \right)$ | (27) |
| --- | --- |

where $E_{min}$ is the stiffness of weak material, $p$ the penalty factor, and $E_{0}$ the stiffness of the element with full material.

The LSF evolution is driven based on nodal sensitivity. Here, the nodal sensitivity $V_{NS}$ is defined as the average sum of the sensitivities of the elements that contain the node, which is given as follows:

| $V_{NS}\boldsymbol{=}\frac{1}{n_{e}}\sum_{1}^{n_{e}} \frac{\partial C}{\partial\rho_{e}}$ | (28) |
| --- | --- |

where $n_{e}$ is the total number of elements that share a common node. We use the gradient-based method to solve the optimization problem. The Lagrangian function for the mathematical model is formed as,

| $L=C+\Lambda\left( \sum_{e=1}^{N_{e}} \rho_{e}v_{e}-A_{f} \right)$ | (29) |
| --- | --- |

where $\Lambda$ is the Lagrangian multiplier. According to the Karush–Kuhn–Tucker (KKT) conditions, by differentiating the Lagrangian function with respect to the design variable, the convergence optimal criteria can be obtained as:

| $\frac{\partial C}{\partial\rho_{e}}+\Lambda=0$ | (30) |
| --- | --- |

Herein, to deduce the LSF evolution governing ODE, we first investigate a more general spatial motion of LSF. Consider a $n$D space, the LSF $\phi\left( \mathbf{X} \right)$ is a function defined in the $n$D space and can propagate in any direction of the $n$D space, where $\mathbf{X=(}x_{1}\boldsymbol{,\cdots}x_{j}\boldsymbol{,\cdots,}x_{n}\mathbf{)}$. Introducing the pseudo time $t$, the zero level-set can be written as,

| $\phi\left( \mathbf{X,}t \right)=0 .$ | (31) |
| --- | --- |

By differentiating the LSF with respect to pseudo time $t$, we have,

| $\frac{\partial\phi}{\partial t}+\frac{\partial\phi}{\partial x_{1}}\frac{\partial x_{1}}{\partial t}+\boldsymbol{\cdots+}\frac{\partial\phi}{\partial x_{j}}\frac{\partial x_{j}}{\partial t}+\boldsymbol{\cdots+}\frac{\partial\phi}{\partial x_{n}}\frac{\partial x_{n}}{\partial t}=0 .$ | (32) |
| --- | --- |

Herein, if $\phi$ is immutable along the $j$th direction in the $n$D space, i.e., ${\partial\phi}/{\partial x_{j}}=0$, then Eq.(32) yields,

| $\frac{\partial\phi}{\partial t}+\frac{\partial\phi}{\partial x_{1}}\frac{\partial x_{1}}{\partial t}+\boldsymbol{\cdots+}\frac{\partial\phi}{\partial x_{j-1}}\frac{\partial x_{j-1}}{\partial t}+\frac{\partial\phi}{\partial x_{j+1}}\frac{\partial x_{j+1}}{\partial t}\boldsymbol{+\cdots+}\frac{\partial\phi}{\partial x_{n}}\frac{\partial x_{n}}{\partial t}=0 .$ | (33) |
| --- | --- |

But if the value of $\phi$ only changes along the $j$th direction in the $n$D space, it has,

| $\frac{\partial\phi}{\partial x_{1}}=\boldsymbol{\cdots=}\frac{\partial\phi}{\partial x_{j-1}}=\frac{\partial\phi}{\partial x_{j+1}}=\boldsymbol{\cdots=}\frac{\partial\phi}{\partial x_{n}}=0 ,$ | (34) |
| --- | --- |

and Eq.(32) becomes,

| $\frac{\partial\phi}{\partial t}+\frac{\partial\phi}{\partial x_{j}}\frac{\partial x_{j}}{\partial t}=0 .$ | (35) |
| --- | --- |

Typically, identify a $3$D space and suppose$\phi\left( \mathbf{X} \right)$ is a function defined in the $3$D space where $\mathbf{X=(}x,y,z\mathbf{)}$. If only the movements along $x$ and $y$ directions deliver changes of $\phi$ in the $n$D space, i.e., ${\partial\phi}/{\partial z}=0$, then Eq.(32) yields,

| $\frac{\partial\phi}{\partial t}+\frac{\partial\phi}{\partial x}\frac{\partial x}{\partial t}\mathbf{+}\frac{\partial\phi}{\partial y}\frac{\partial y}{\partial t}=0 ,$ | (36) |
| --- | --- |

which is the classic conventional Hamilton-Jacobi (H-J) partial derivative equation (PDE) for a 2D design problem where the structural domain is located in the $x$-$y$ plane.

Now let us change the way of movement of $\phi$, e.g., assuming $\phi$ is only changeable just along the $z$ direction (i.e., ${\partial\phi}/{\partial x}=0$ and ${\partial\phi}/{\partial y}=0$), we have,

| $\frac{\partial\phi}{\partial t}+\frac{\partial\phi}{\partial z}\frac{\partial z}{\partial t}=0 .$ | (37) |
| --- | --- |

As can be learned, the update of LSF is only determined by information of $z$ direction. Particularly, if ${\partial\phi}/{\partial z}$ is a constant, the PDE of Eq.(37) becomes an ODE. We define the update information as velocity $V_{z}$, and we obtain,

| $\frac{d\phi}{dt}+V_{z}=0 ,$ | (38) |
| --- | --- |

Herein, the Eq.(38) is borrowed to update LSF in this paper and after discretization of the design domain the nodal sensitivity is used to drive the propagation of LSF, i.e.,

| $\frac{d\phi}{dt}+V_{NS}=0 .$ | (39) |
| --- | --- |

Thus, the ODE for the evolution of LSF is formulated (note here that Eq.(38) is established for any arbitrary point in the design domain and it is naturally applicable for all analysis mesh nodes). With the introduction of nodal sensitivity, the evolution of LSF can be used for the optimization of the structure. However, the volume constraint and convergence evaluation are not incorporated in Eq.(39). Considering the optimal criteria, it is easy to prove that the optimal criteria for elements is equivalent for nodes, hence, based on the elemental optimal criteria in Eq.(30), the nodal optimal criteria can be attained as follows,

| $V_{NS}+\Lambda=0 .$ | (40) |
| --- | --- |

Replace $V_{NS}$ in Eq.(39) with $V_{NS}+\Lambda$, we have,

| $\frac{d\phi}{dt}\mathbf{+}\left( V_{NS}+\Lambda\right)\mathbf{=}0\mathbf{.}$ | (41) |
| --- | --- |

Eq.(41) delivers such a message that the Lagrangian multiplier $\Lambda$ is introduced to tackle material volume constraint, and the LSF stops propagating as the optimal criteria term $\left( V_{NS}+\Lambda\right)$ becomes $0$, in other words, theoretically, the evolution of LSF ends up with all nodal sensitivities equal to the same constant of Lagrangian multiplier, which is a KKT point. Thus, based on Eq.(41), the iterative update format of LSF can be developed as follows,

| $\phi_{i+1}=\phi_{i}-\Delta t\left( V_{NS}+\Lambda\right) ,$ | (42) |
| --- | --- |

where $i$ is the iteration number and $\Delta t$ is the chosen time step. The stopping criteria for the optimization can be the minimum change of the objective function $R$ (compared with, e.g., the last five iterations), or a user-defined maximum loop, i.e.,

| $\frac{\left\vert R_{i}\boldsymbol{-}R_{i-5} \right\vert}{R_{i}}<1\times{10}^{-4} ,\mathrm{or}i>\mathrm{loop}_{max} .$ | (43) |
| --- | --- |

As optimization goes by, though the LSF will stop varying based on the convergence criteria of Eq.(30), the value of $\phi$ can be very large. In some cases, this situation may suppress the topological changing ability, and the optimization is more likely to fall into local optimum or ill-posed solutions owing to that the value of $\phi$ is limited to change along the direction perpendicular to the structure interface. As a result, the topological variations, such as structural formation, disappearance, translation, rotation, merging, and so on, are achieved simply based on the LSF $\phi$ to grow above or below the cutting level-set, which is different from conventional level-set methods in which the LSF is evolved based on normal perturbations of boundaries. Here, to counter numerical issues during the optimization process, we propose a regularization scheme as follows,

| $\phi^{*}=\alpha\phi, 0<\alpha<1$ | (44) |
| --- | --- |

where $\alpha$ is a positive scaling factor. The regularization is a linear scaling manner, which works as a re-initialization strategy. By periodically re-initializing the level-set function, the topological defects can be naturally, effectively, and efficiently eliminated, making the optimization more active and responsive.

We adopt the full material design as the initial design to perform optimization as it can ensure broader design space. The given material area $G_{i}$ at each iteration step is linearly relaxed from the initial full material design, which takes the following form,

| $G_{i}=A_{0}-\left( A_{0}-A_{f} \right)\frac{i}{N_{R}} i\leq N_{R}$ | (45) |
| --- | --- |

where $A_{0}$ is the initial material area fraction equal to 1 when choosing the initial design with full material, $N_{R}$ the prescribed relaxation steps to control material area variation.

For the case of minimization of compliance, the derivative of the objective function with respect to the design variable can be written as,

| $\frac{\partial C}{\partial\rho_{e}}=-p\left( \rho_{e} \right)^{p-1}\mathbf{u}_{e}^{T}\mathbf{K}_{e}\mathbf{u}_{e}$ | (46) |
| --- | --- |

**2.2 Optimization process**

The flowchart for the optimization algorithm is illustrated in **Supplementary Fig. 2a**. The basic procedures for the optimization include preparation, optimization, and postprocess. In the preparation part, the design variable, design domain, objective, constraints, material property, algorithm parameter, and boundary condition are defined, and the design domain is meshed. In the optimization part, the optimization problem is iteratively solved and the structural design is updated. Finally, the converged design is subjected to postprocess.

**2.3 Boundary condition**

As the multilayer configurations cause complexity in structural boundaries, the boundary conditions need to be identified clearly. To fully exploit the symmetry of the initial design configuration of a unit cell to save computation cost, the 1/8 divisions for the initial designs are applied to perform optimization. For example, **Supplementary Fig. 2b** illustrates the boundary condition definition for the Schwarz P-set under uniaxial loading. Three types of boundary conditions are identified, including the displacement boundary, force boundary, and periodic boundary. For a unit cell, the periodic boundary is replaced with the free boundary.

**Supplementary Note 3: Practical implementation**

To carry out the whole modeling, optimization, and numerical simulation, a series of tools are employed in practical implementation. As demonstrated in **Supplementary Fig. 2c**, we first use MATLAB to generate the candidate cell surface according to the analytical expression and then construct the initial multilayer design, which is a ‘.stl’ document. Then the geometrical document is obtained based on the ‘.stl’ document. Then the geometrical document is meshed in HYPERMESH. Note here the mesh needs to be fine enough to ensure sufficient optimization design space since the design variable density is mesh element-based. After that, the topology optimization is carried out in MATLAB and optimized results are attained, using our homemade code based on the proposed ODE-driven level-set density method. Again, the optimized ‘.stl’ document is remodeled and the geometrical model is meshed. Here, the mesh for the optimized model does not have to be too dense. On the contrary, a coarse mesh helps save computation costs with sufficient proficiency for numerical simulation and verification, which are implemented in commercial software ABAQUS and COMSOL.

**Supplementary Note 4: Method verification**

To ensure the correctness and effectiveness of the whole method, several analysis techniques need to be verified. In the physical field analysis part of topology optimization, the hybrid element combining membrane element with DKQ plate element is applied to perform the finite element analysis, and the accuracy is verified in comparison with the ABAQUS results. As demonstrated in **Supplementary Fig. 3a**, for the same benchmark case, the offset of our result from the ABAQUS result is 0.086%.

The static simulation with periodic boundary conditions was applied to calculate the elastic constants. Also, the quasistatic compressing method was used to study the constitutive relationships of the unit cells. The material iron (Young’s modulus: 210GPa, yield strength: 400MPa) is assigned to all unit cells. Both static and quasistatic methods are applied to calibrate the material’s constitute properties. As displayed in **Supplementary Fig. 3b** and **Supplementary Fig. 3c**, the calculated results are consistent with the given material properties, certifying the validity of the analysis method.

We study shell-based lattice at low density in this work. The precision and effectiveness of the analysis element are verified. The stress-strain constitutive relationships at different densities are investigated with both shell and solid elements. As compared in **Supplementary Fig. 3d**, the offset of results from shell element and solid element is negligible at low densities, but shows an enlarging trend as the relative density increases (with increasing component thickness). Therefore, the shell element can ensure sufficient accuracy for lattices at low densities.

The mesh convergence is verified. As shown in **Supplementary Fig. 3e**, four meshes with different densities are examined for the same benchmark problem, and the calculated results basically show the same with each other.

**Supplementary Note 5: Optimization anomaly**

The so-called optimization might produce results not better than the original structure. This anomaly is due to several factors. For optimized shell components, in comparison to the original model without optimization, we use the thickness compensation scheme to ensure they are at the same relative density for fair comparison. The thickness-compensation, however, does not necessarily guarantee that the thickened optimized results are better than the original models. This is because the optimization of shell structures is thickness-dependent. For example, as can be seen in **Supplementary Fig. 6a**, the optimized results show quite different topological configurations as the thickness changes for the Neovius model. Thus, the optimized result with compensated thickness can be different from the optimized result with the original thickness. On the other hand, topology optimization reconfigures the material distribution, which may lead to a shift of deformation mode, such as the change from stretching-dominated to bending-dominated. This may weaken the mechanical performance of the original model. For example, as can be seen in **Supplementary Fig. 6b**, the optimized IWP shows clear bending behavior while the Neovius is still stretching-dominated. As a result, the thickness-compensation works well for the Neovius and its stiffness demonstrates remarkable improvement, while the performance of IWP may suffer deterioration. In addition, topology optimization inevitably creates holes on the surface of the designable region (though we can control the area fraction to limit the area of those holes), as we use shell element to optimize and there is only one layer of the shell element, which can induce stress concentration and local deformation around those holes (**Supplementary Fig. 6c**), thereby significantly impairing the mechanical performance.

To counter the above-mentioned limitations, one way is to use the multilayer solid element to perform the optimization (**Supplementary Fig. 6d**). Among the multilayer solid elements, each layer can be assigned as designable or non-designable. In this way, the optimization can be implemented without creating holes by deliberately prescribing a non-designable region, thereby maintaining the smooth surface and avoiding stress concentration. Although this may render close-cell of the cellular material, leading to difficulty in printing postprocess, the setting for non-designable regions can always be optional.

**Supplementary Note 6: Disparity between experiment and simulation**

In the physical experimental part, we aimed to test the mechanical properties of the printed models. We only study the relative proportional ratios of Young’s moduli of the P set and the Opt-P set, which means that the experimental results should be material-independent. Thus, we chose four different printing materials to verify the simulation results. In our numerical simulation, the material iron is used (Young’s modulus: 210GPa, yield strength: 400MPa), and the material properties are verified using both static (**Supplementary Fig. 3b**) and dynamic methods (**Supplementary Fig. 3c**). Here, different printing materials have different mechanical properties, plastic, brittle, or quasi-brittle. The yield strength can vary for different materials. Therefore, we only consider the effective Young’s moduli of the printed models with the different materials, i.e., the stiffness, which can be obtained by calculating the slope of the linear part of the constitutional engineering stress-strain curve (**Supplementary Fig. 14**). **Supplementary Fig. 16** displays the printed cell models and their mass comparison. **Supplementary Table 3** summarizes the effective Young’s moduli of the printed cell models. **Supplementary Table 4** presents the percentages of effective Young’s moduli of the printed cell models normalized by the result of P-1. Different printing materials may result in different normalized values, but the tendency is consistent with the simulation results. As can be seen in **Supplementary Table 4**, the relative proportional ratios among the P set and Opt-P set for the non-metal model (TPU and PA12) show larger values as a whole, but the variation tendency compared with the normalized P-1 is consistent. It seems that the variance between the experiment and the simulation is a proportional factor. For example, if the effective Young’s moduli of the TPU result (except P-1) multiply a proportional factor of 75.3%, we obtain results very close to the numerical simulation results (**Supplementary Table 5**). If the effective Young’s moduli of the PA12 result (except P-1) multiply a proportional factor of 52.3%, we obtain results very close to the numerical simulation results (**Supplementary Table 5**). For the metal result (SS316, AlSi10Mg), the relative proportional ratios show smaller values for P-4, -5 and Opt-P-4, -5. The variances are mainly caused by the printing technics and prototyping quality ^[14]^. For the metal printing technology that requires high temperatures, the residue stress from cooling down from high temperatures may lead to many small defects and cracks, as a result, weakening the mechanical properties of the printed model ^[14]^. For cell models, the local printing quality may affect the experimental results to some extent. For the scale of the 4X4X4 case, the local defects can be alleviated significantly (**Supplementary Fig. 15**). **Supplementary Fig. 17** displays the printed 4X4X4 models and their mass comparison. **Supplementary Table 6** and **Supplementary Table 7** summarize the effective Young’s moduli of the printed 4X4X4 models with the materials of PA12 and AlSi10Mg, respectively. **Supplementary Table 8** presents the percentages of effective Young’s moduli of the printed 4X4X4 models normalized by the result of P-1. For the PA12 model, the printed scale is 120mmX120mmX120mm. Such length-scale can ensure better printing quality (**Supplementary Fig. 18a**). As a result, the experimental results are consistent with corresponding simulation results (**Supplementary Table 8**). The FBC-Opt-P-2 model is an exception because the mass loss (around 27.4g (17.8%) compared with the P-1) is significant after removing the support structures and powder remainder (**Supplementary Fig. 17c**). For the AlSi10Mg model, we printed the metal models with the scale of 60mmX60mmX60mm, and the printing quality is relatively lower (**Supplementary Fig. 18b**). Again, the metal results (AlSi10Mg) of Opt-P-4 and -5 cannot reach the simulation level due to the reason of the printing technics itself and prototyping quality (**Supplementary Table 8**).


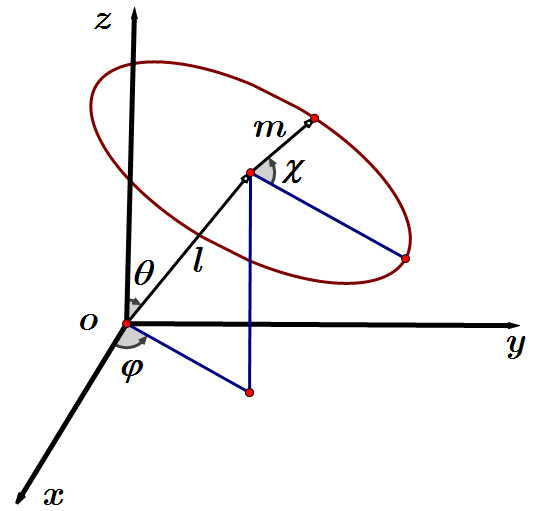


**Supplementary Fig. 1 | Young's modulus and shear modulus in in an arbitrary direction**

**
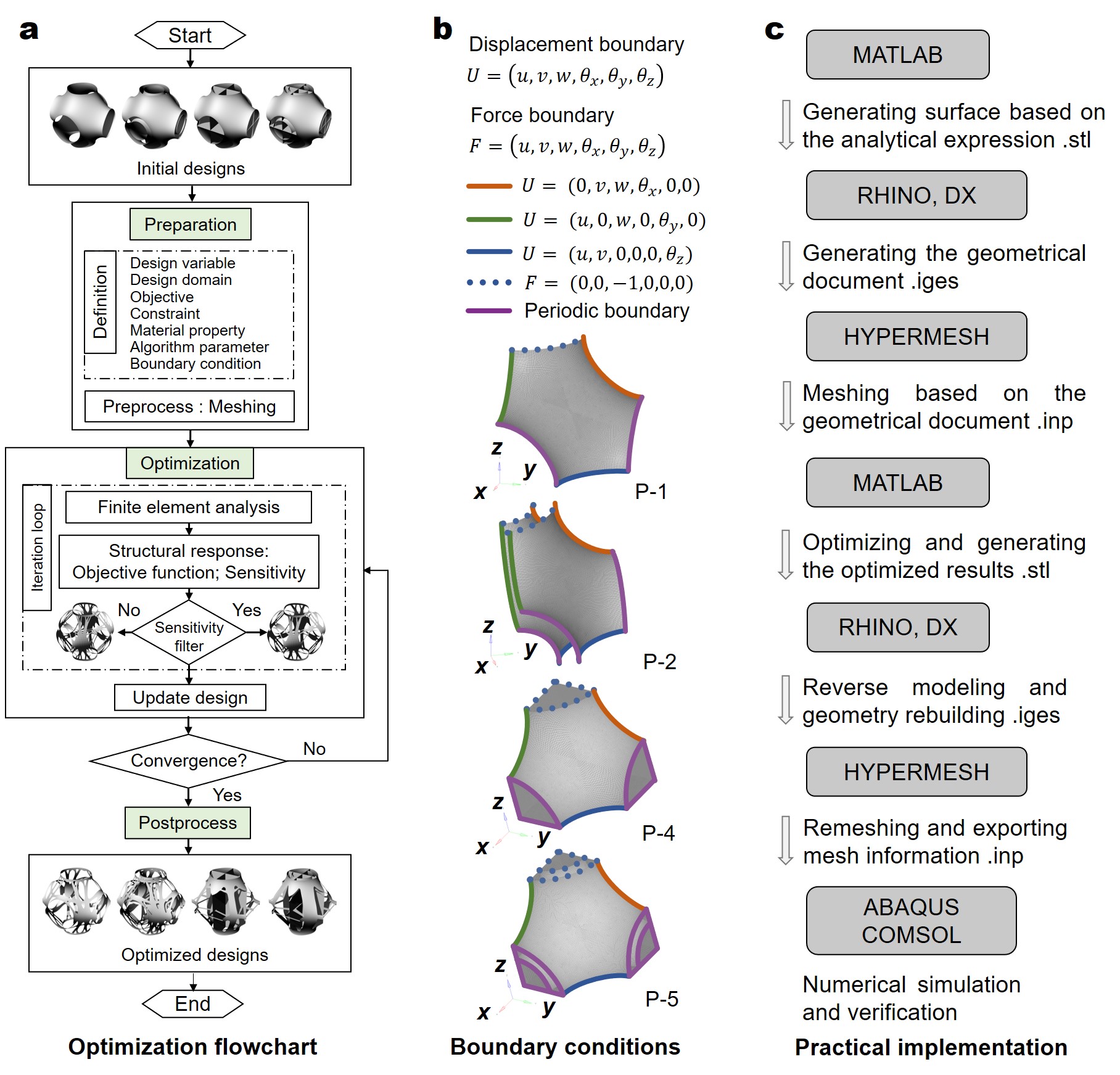
**

**Supplementary Fig. 2** **| Design and optimization strategy for multilayer shell-based lattice.** **a**, optimization flowchart. **b**, definition of boundary conditions. **c**, practical implementation.


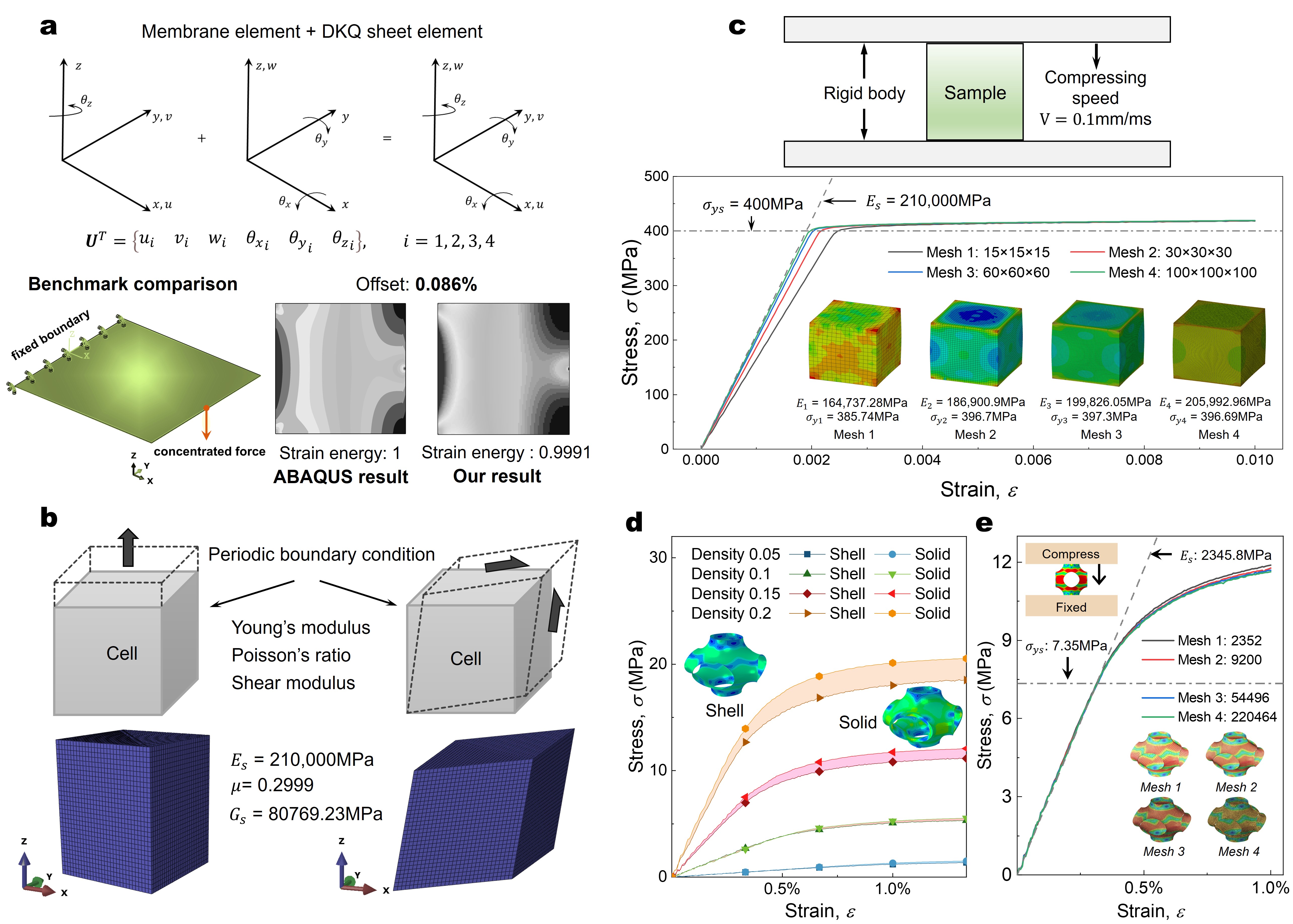


**Supplementary Fig. 3 | Method verification.** **a**, verification of element precision. **b**, verification of material property through static analysis method. **c**, verification of material property through quasistatic compressing method based on explicit dynamic computation. **d**, verification of element type. **e**, verification of mesh convergence. Source data are provided as a Source Data file.


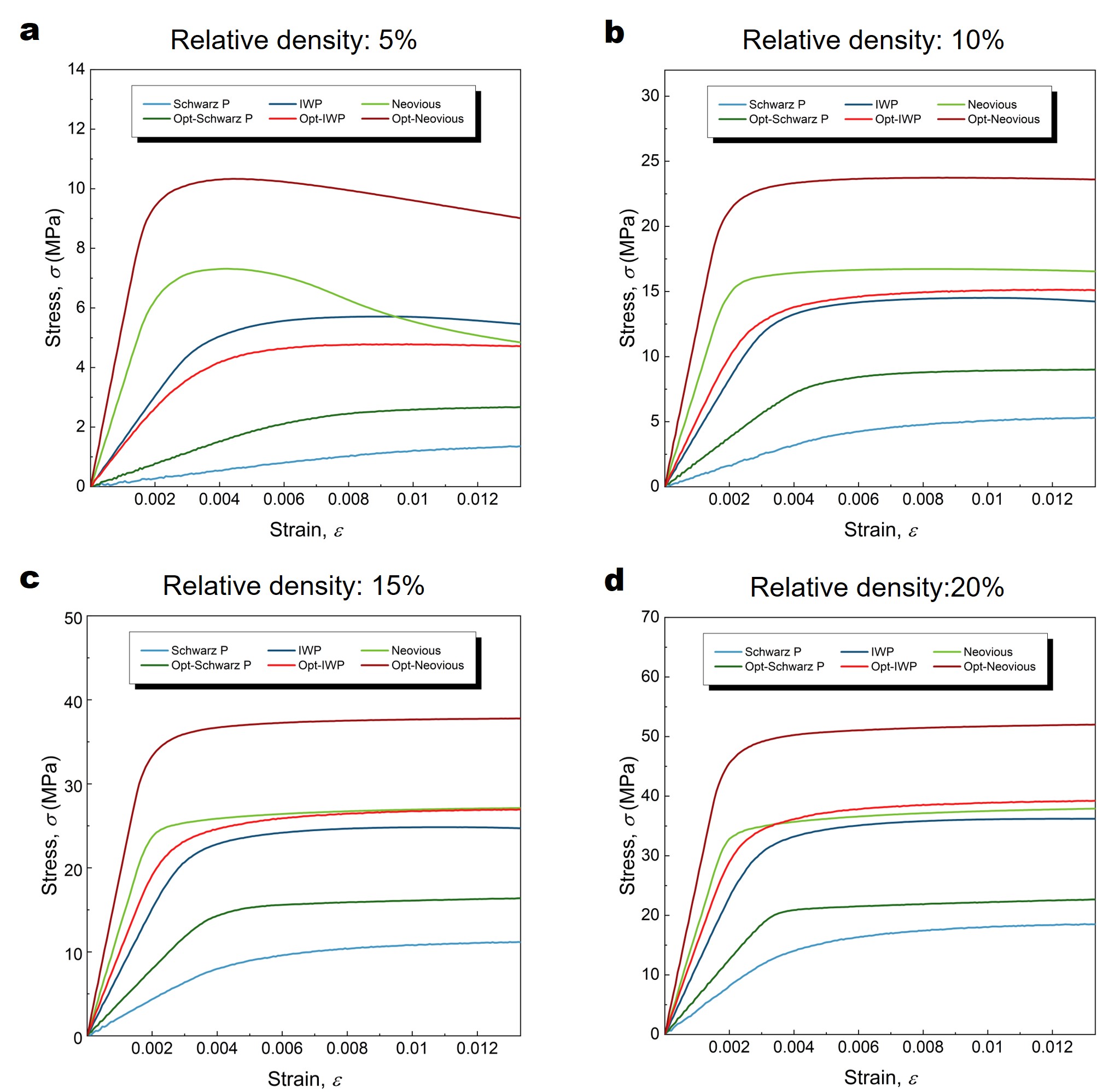


**Supplementary Fig. 4 | Stress-strain curves for Schwarz P, IWP, Neovius, and their optimized results with different relative densities**. **a**-**d**, stress-strain curves for relative density 5%, 10%, 15%, and 20%, respectively. Source data are provided as a Source Data file.


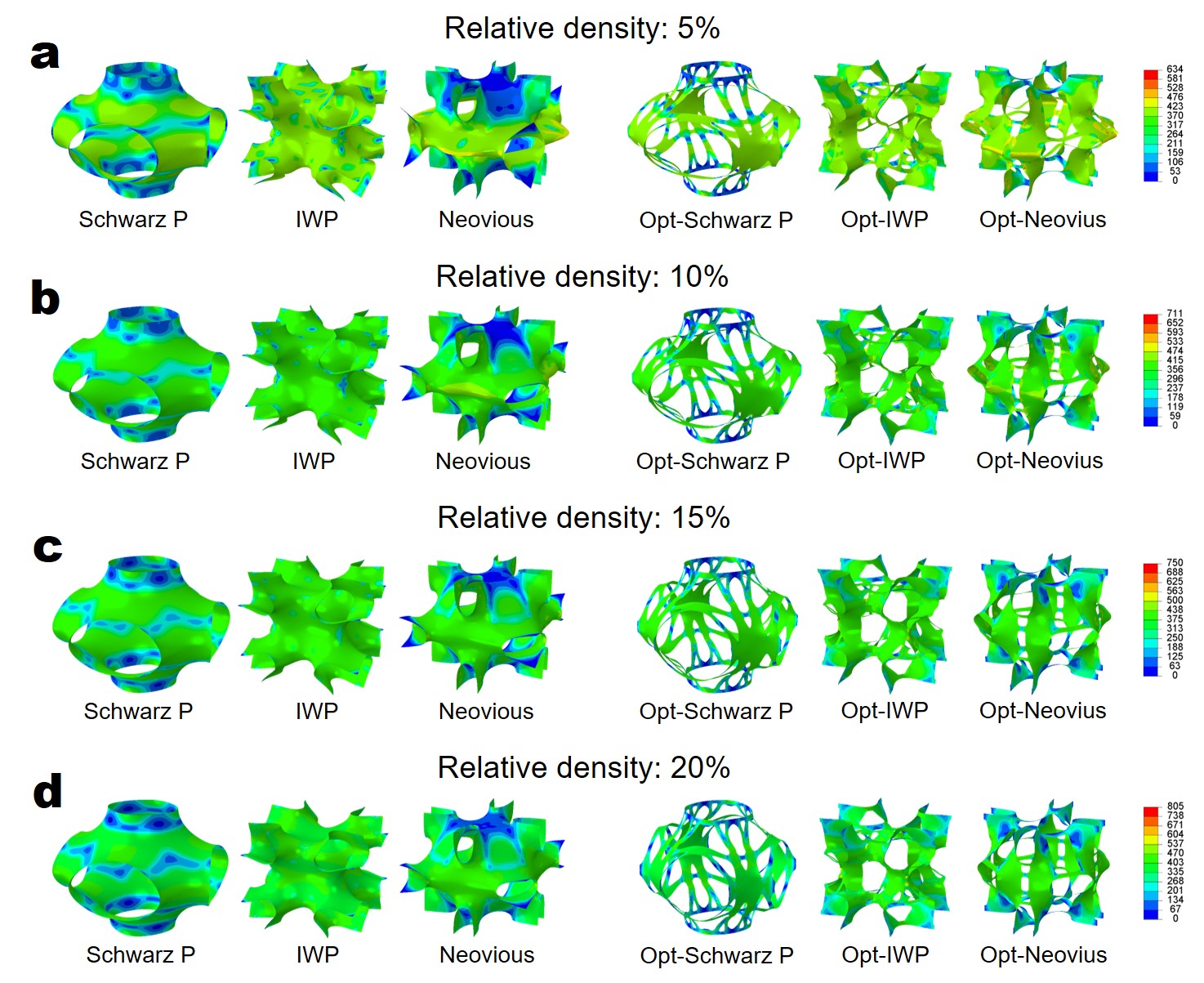


**Supplementary Fig. 5 | Uniaxial deformation for Schwarz P, IWP, Neovius, and their optimized results with different relative densities**. **a**-**d**, uniaxial deformation for relative density 5%, 10%, 15%, and 20%, respectively.


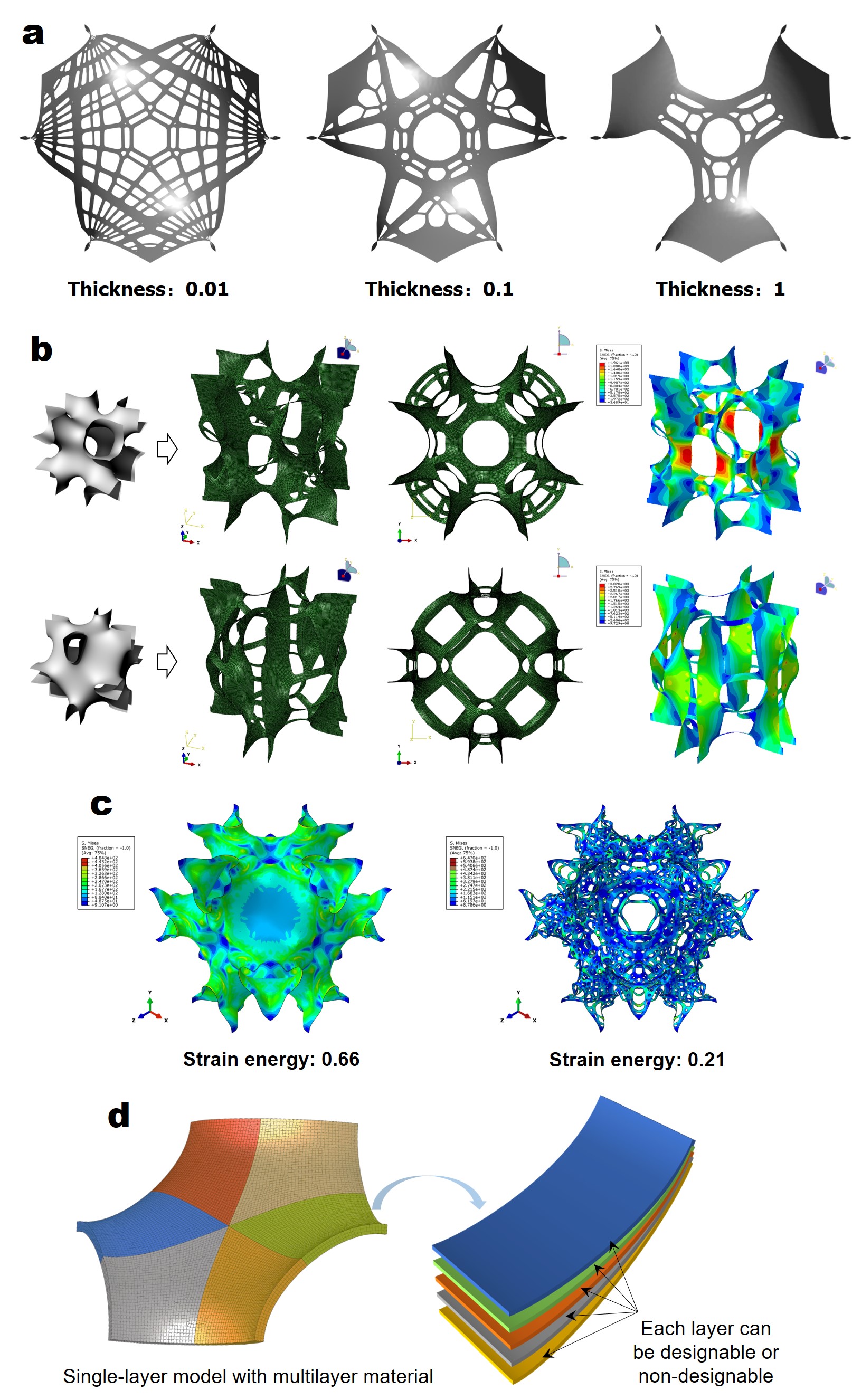


**Supplementary Fig. 6 | Uniaxial deformation for Schwarz P, IWP, Neovius, and their optimized results with different relative densities**. **a**-**d**, uniaxial deformation for relative density 5%, 10%, 15%, and 20%, respectively.


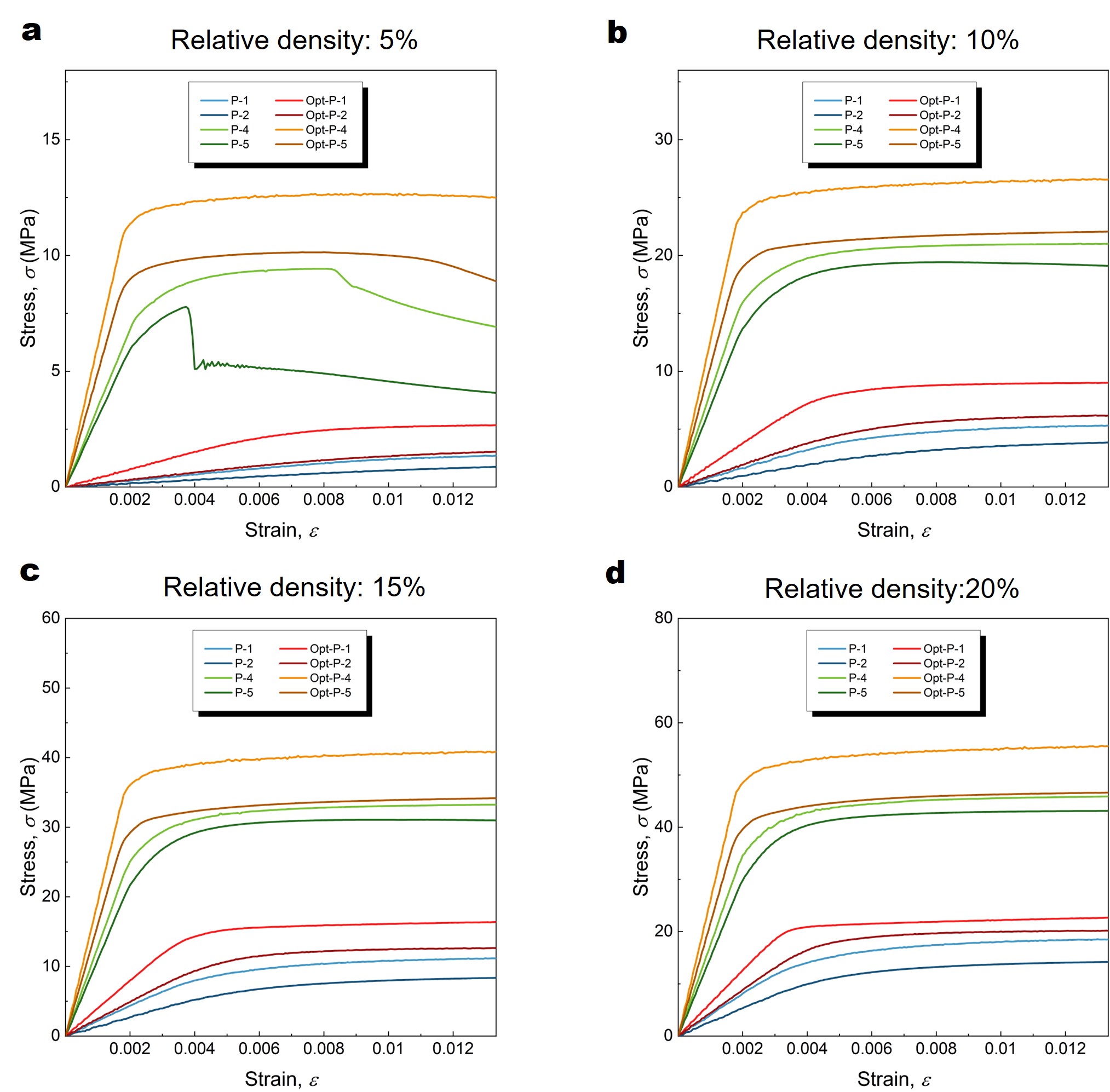


**Supplementary Fig. 7 | Stress-strain curves for Schwarz P and its multilayer variants and their optimized results under uniaxial loading with different relative densities**. **a**-**d**, stress-strain curves for relative density 5%, 10%, 15%, and 20%, respectively. Source data are provided as a Source Data file.


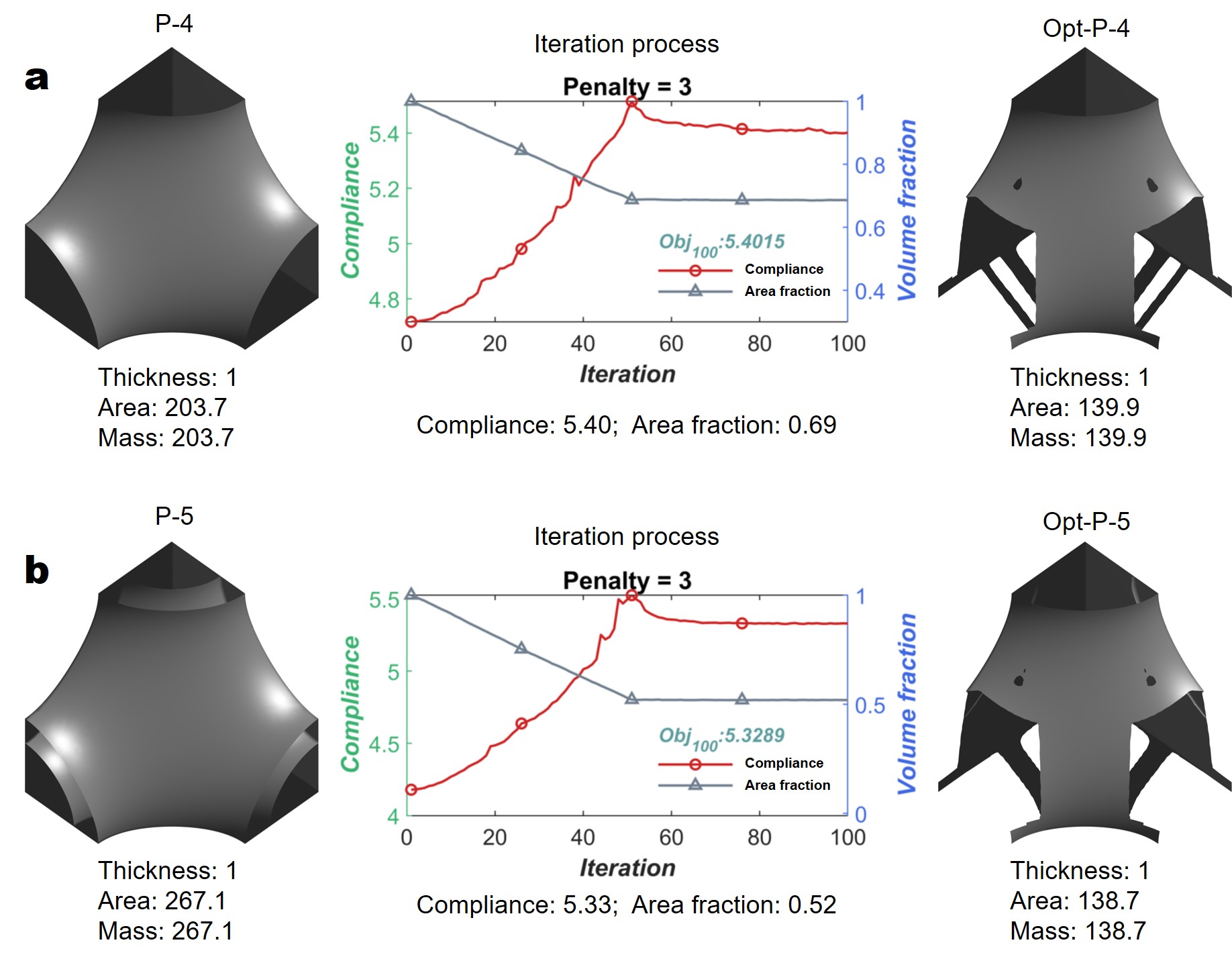


**Supplementary Fig. 8 | Optimization comparison for P-4 and P-5**. **a**, optimization for P-4. **b**, optimization for P-5.


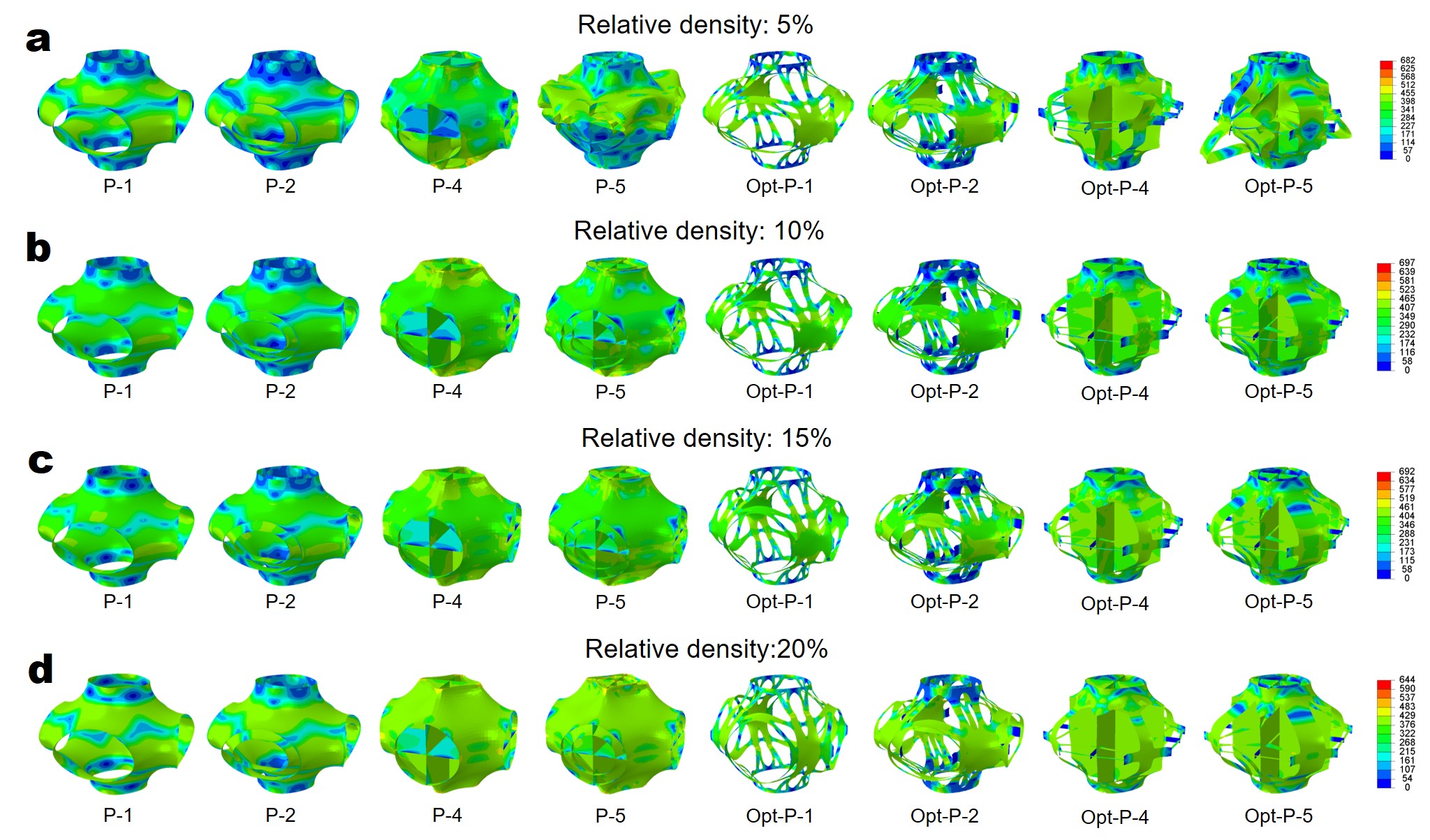


**Supplementary Fig. 9 | Uniaxial deformation for the P set and their optimized results with different relative densities**. **a**-**d**, uniaxial deformation for relative density 5%, 10%, 15%, and 20%, respectively.


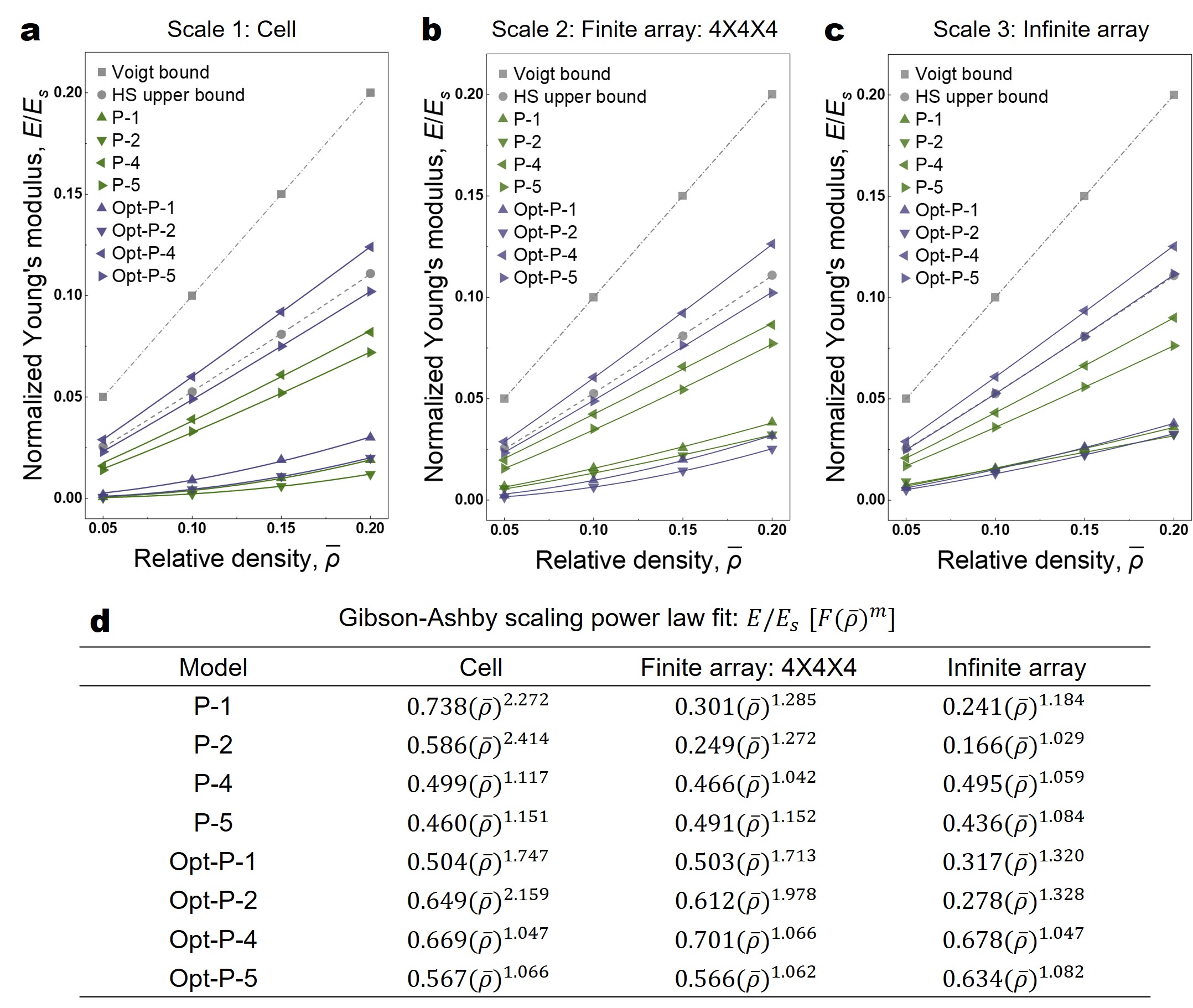


**Supplementary Fig. 10 | Scale effect**. **a~c**, normalized Young’s moduli versus relative density for the cell, finite array, and infinite array scales, respectively. **d**, comparison of Gibson-Ashby scaling power law fit for the Schwarz P set and their optimized results with different scales. Source data are provided as a Source Data file.


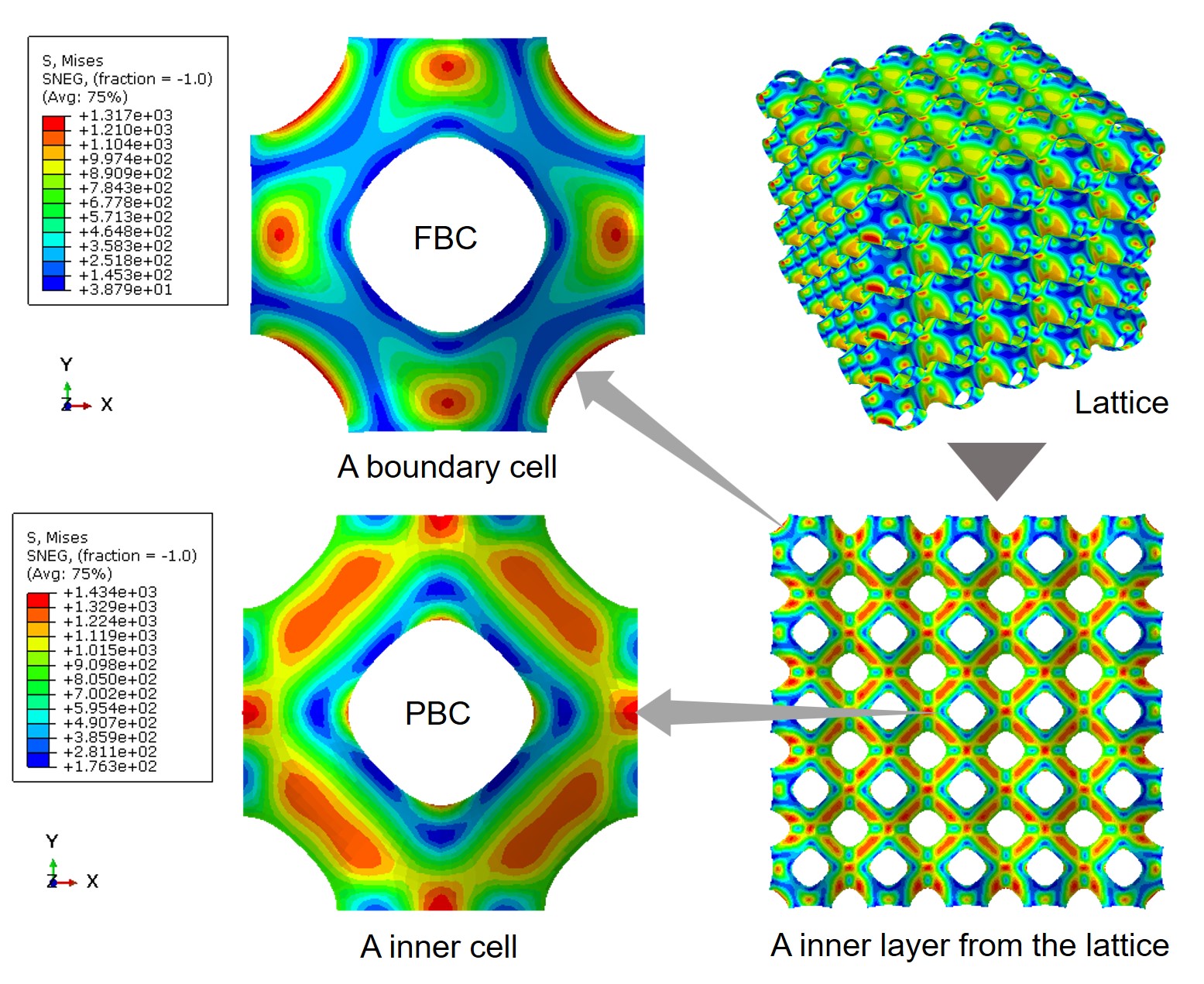


**Supplementary Fig. 11 | Comparison of different deformation modes for different cells inside a lattice.** The boundary condition for a boundary cell is akin to the cell with free boundary condition The boundary condition for a inner cell is akin to the cell with periodic boundary condition.


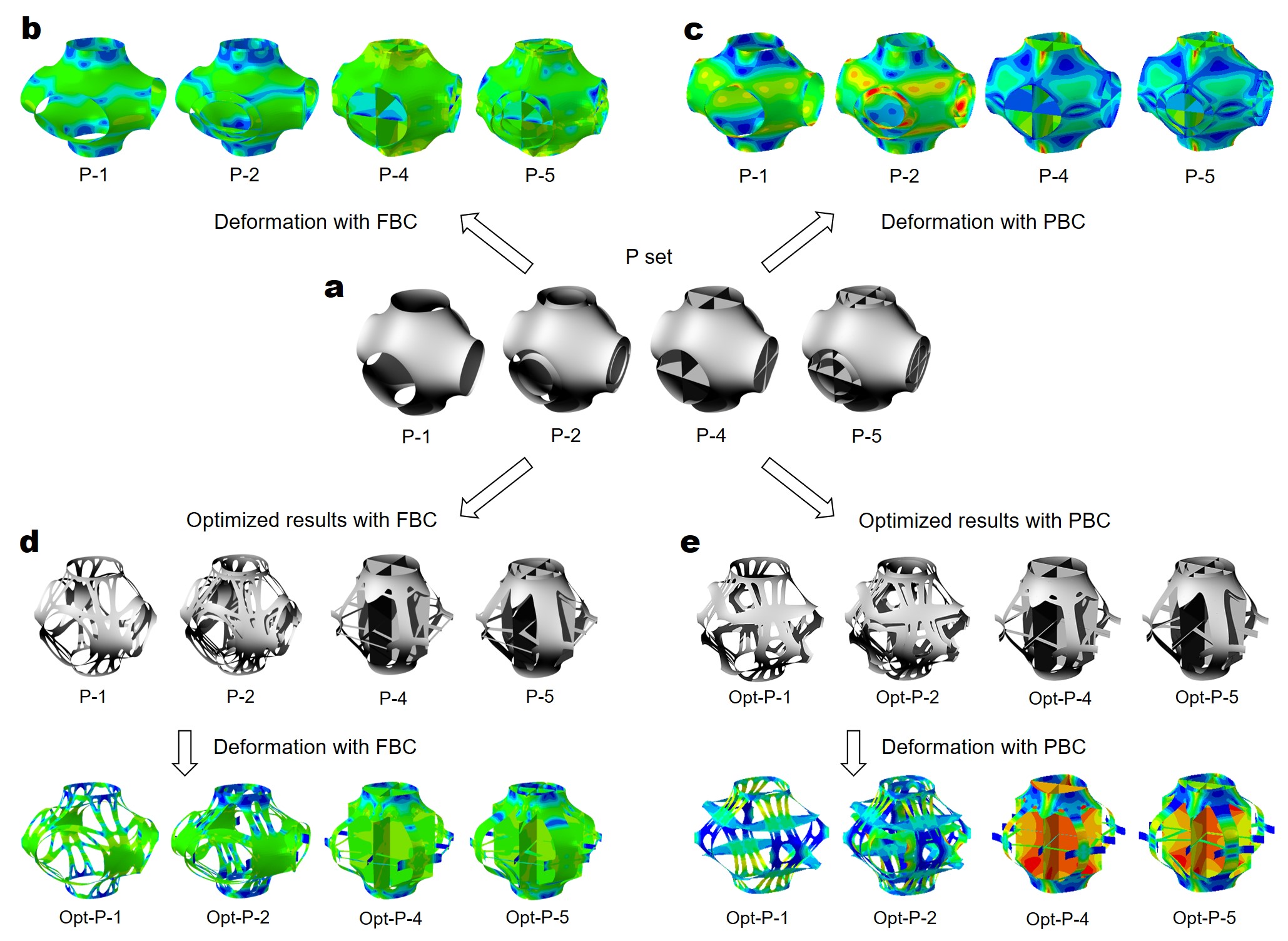


**Supplementary Fig. 12 | Comparison of optimized results with FBC and PBC.** **a**, original Schwarz P set. Subfigures (**b**) and (**c**) compare the deformation modes of Schwarz P set with FBC and PBC, respectively. Subfigures (**d**) and (**e**) compare the optimized results and deformation modes of Schwarz P set with FBC and PBC, respectively.


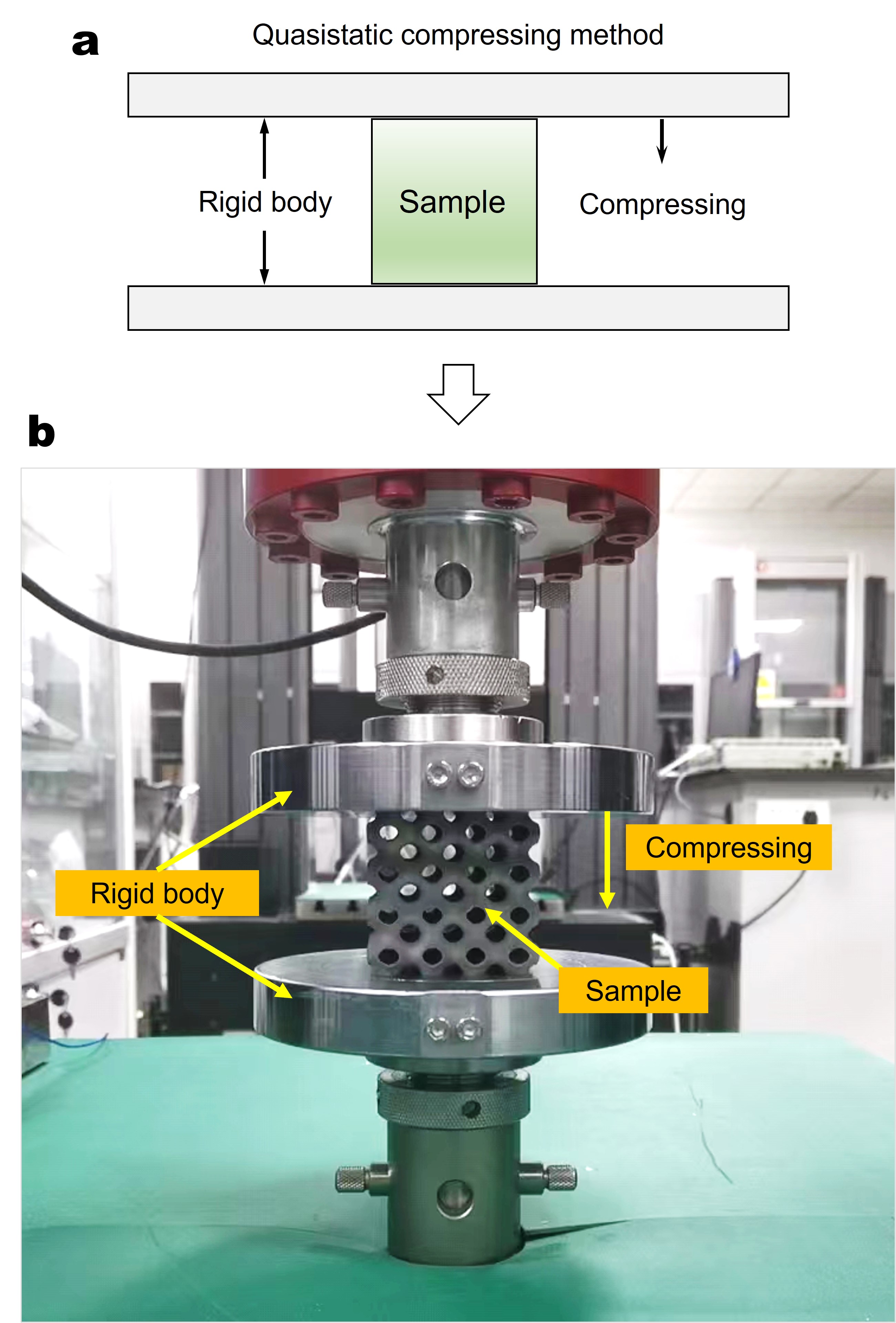


**Supplementary Fig. 13 | Physical situ compressing experiment**. **a,** illustration for the quasistatic compressing method. **b,** situ compressing using a universal testing machine.


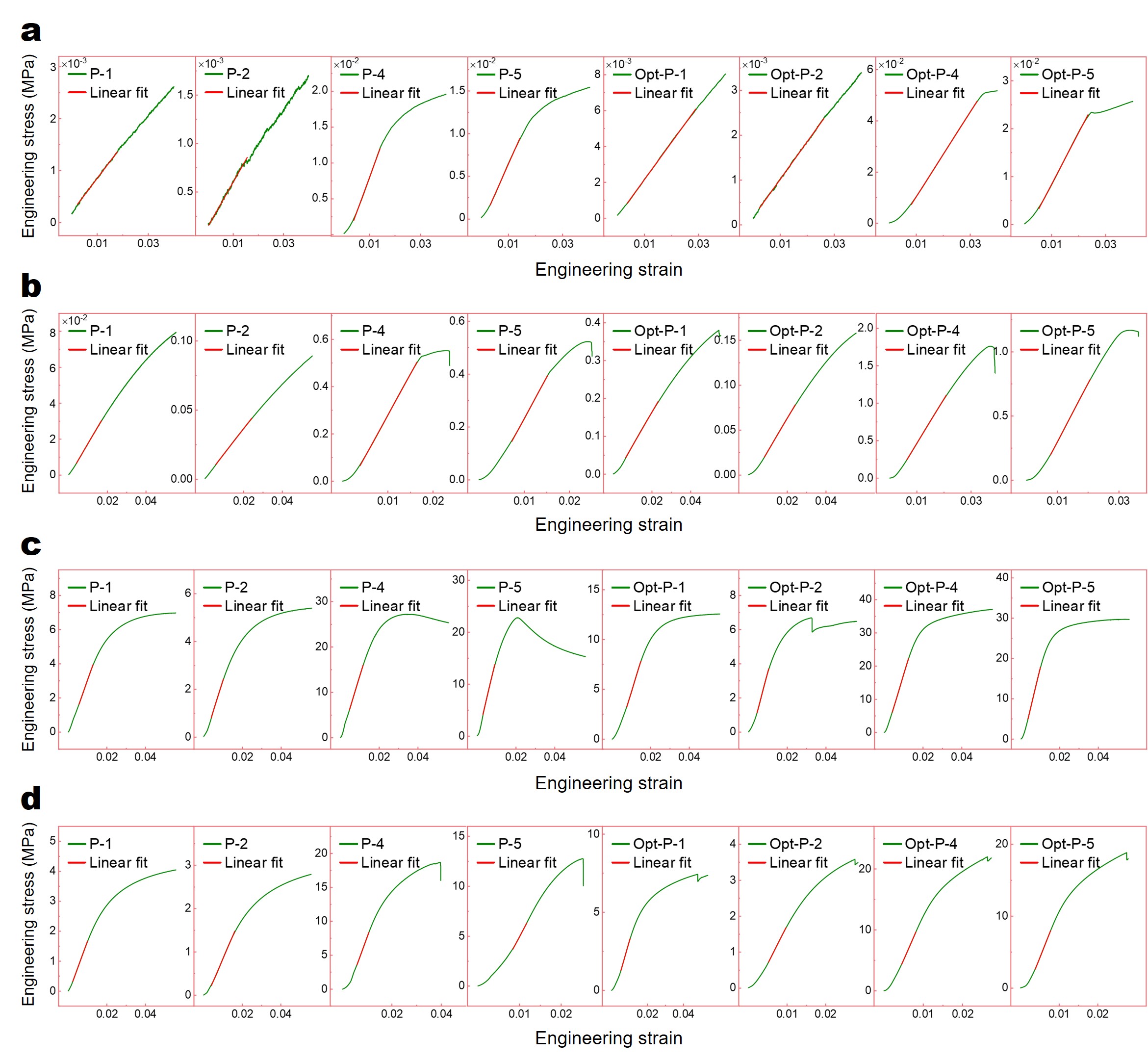


**Supplementary Fig. 14 | Linear fit for the effective Young’s moduli of the cell models with different materials.** **a**, TPU. **b**, PA12. **c**, SS316. **d**, AlSi10Mg. Source data are provided as a Source Data file.


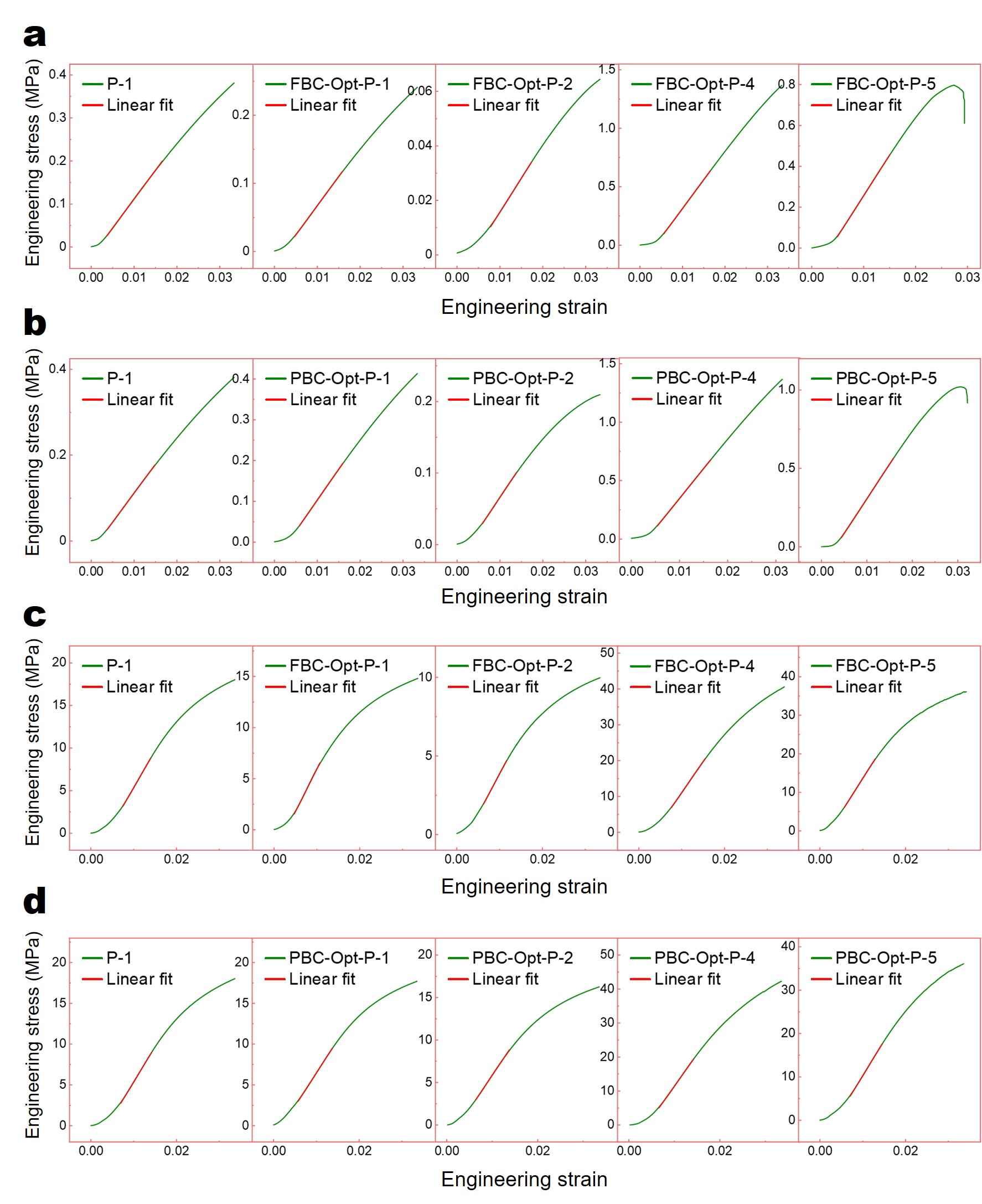


**Supplementary Fig. 15 | Linear fit for the effective Young’s moduli of the 4X4X4 models with different materials.** **a**, PA12 (FBC). **b**, PA12 (PBC). **c**, AlSi10Mg (FBC). **d**, AlSi10Mg (PBC). Source data are provided as a Source Data file.


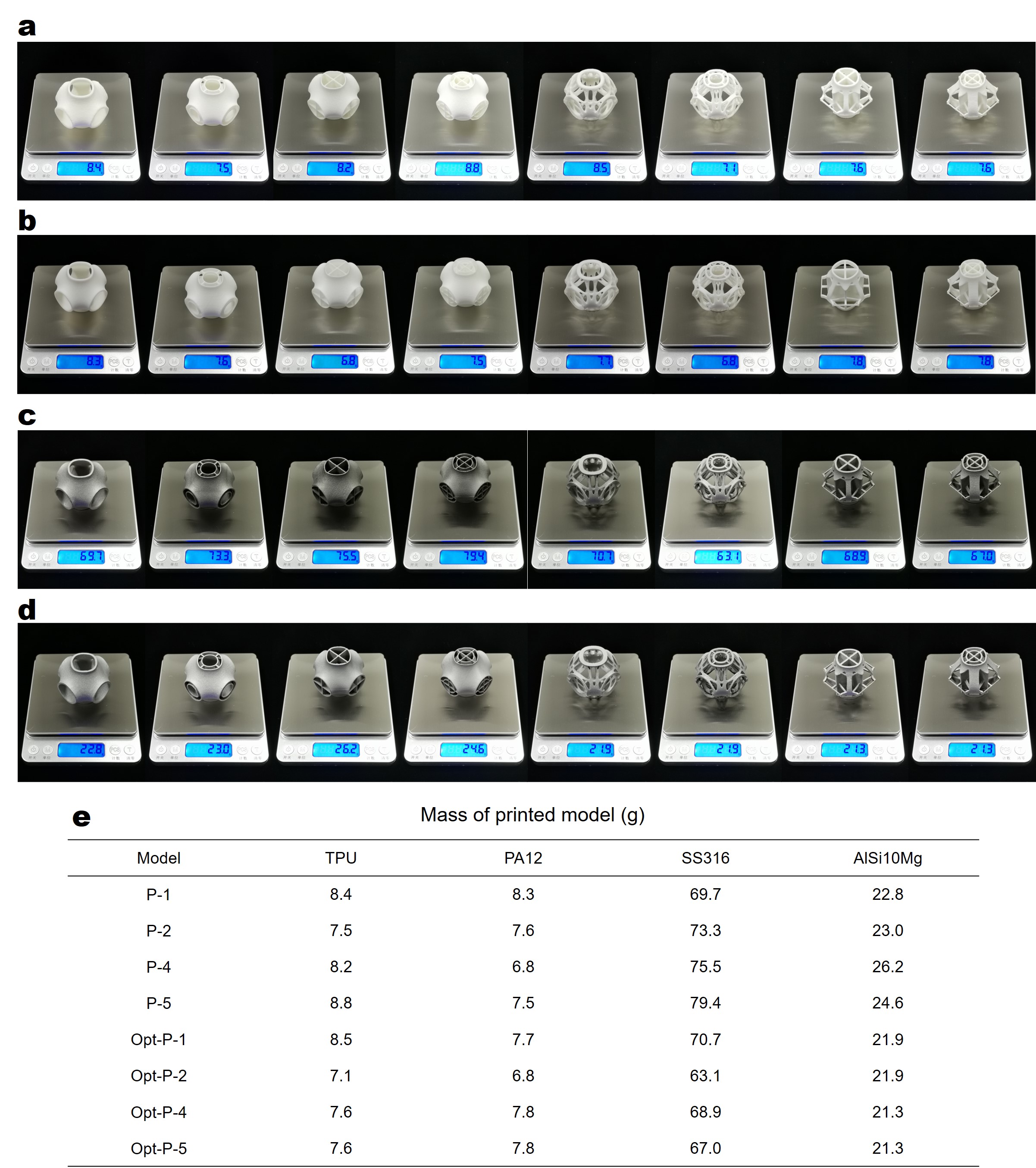


**Supplementary Fig. 16 | Fabricated cell models and mass comparison**. **a**-**d**, printed cell models with TPU, PA12, SS316, and AlSi10Mg, respectively. **d**, comparison of mass with different materials. **e**, comparison of mass with different materials.


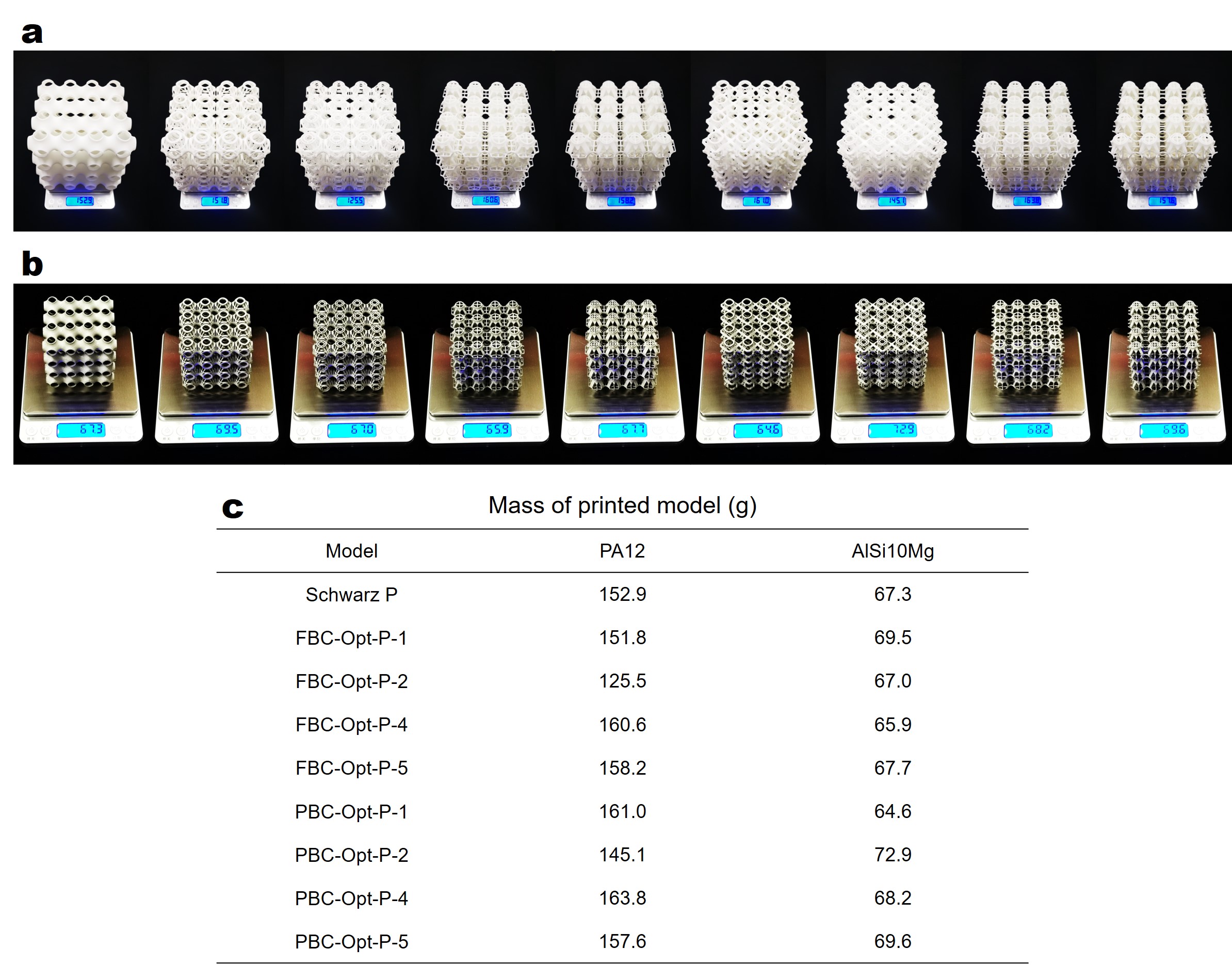


**Supplementary Fig. 17 | Fabricated 4X4X4 array models and mass comparison. a**-**b**, printed 4X4X4 array models with PA12 and AlSi10Mg, respectively. **c**, comparison of mass with different materials.


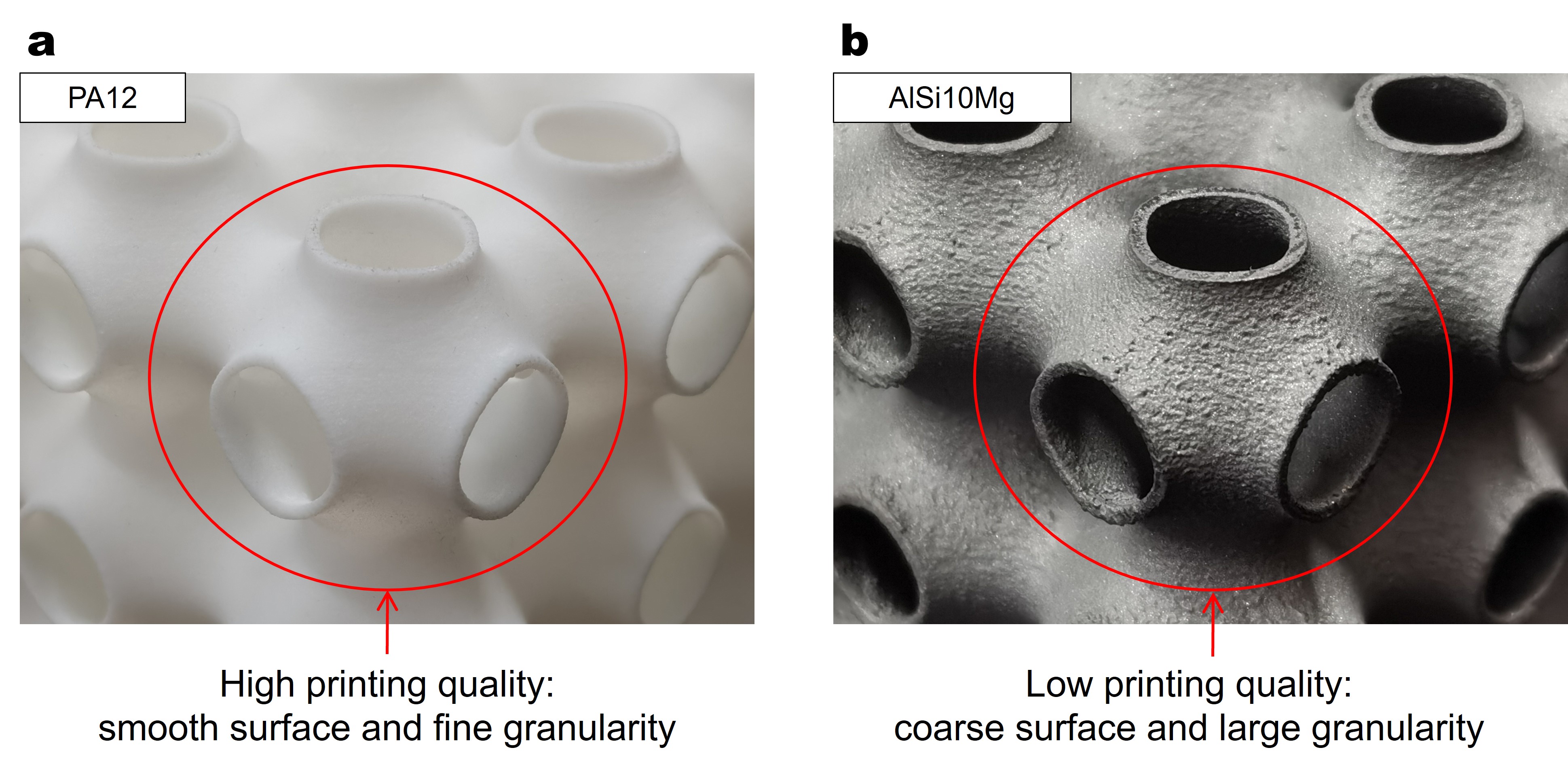


**Supplementary Fig. 18 | Printing quality comparison for 4X4X4 array models fabricated with PA12 and AlSi10Mg. a**, printed models with PA12. **b**, printed models with AlSi10Mg.

**Supplementary Table 1** Young’s moduli and yield strengths comparison for different single-layer based models (MPa)

| Model | Relative density | | | | | | | |
| --- | --- | --- | --- | --- | --- | --- | --- | --- |
|  | 5% | | 10% | | 15% | | 20% | |
|  | Young’s  modulus | Yield strength | Young’s  modulus | Yield strength | Young’s  modulus | Yield strength | Young’s  modulus | Yield strength |
| Schwarz P | 139.34 | 1.05 | 813.24 | 3.89 | 2128.1 | 7.54 | 4038.9 | 12.35 |
| IWP | 1495.9 | 3.99 | 4121.9 | 10.77 | 7569 | 18.94 | 11504 | 25.64 |
| Neovius | 3391.5 | 5.43 | 8003 | 13.61 | 12727 | 21.79 | 17799 | 30.37 |
| Opt-Schwarz P | 381.98 | 1.92 | 1880.3 | 7.01 | 3967.3 | 19.11 | 6304.9 | 27.54 |
| Opt-IWP | 1336.7 | 3.01 | 4975.5 | 10.25 | 9820.1 | 19.11 | 15191 | 27.54 |
| Opt-Neovius | 5496.6 | 8.51 | 12167 | 19.1 | 18938 | 29.98 | 25815 | 39.44 |

**Supplementary Table 2** Young’s moduli and yield strengths comparison for the P set and Opt-P set (MPa)

| Model | Relative density | | | | | | | |
| --- | --- | --- | --- | --- | --- | --- | --- | --- |
|  | 5% | | 10% | | 15% | | 20% | |
|  | Young’s  modulus | Yield strength | Young’s  modulus | Yield strength | Young’s  modulus | Yield strength | Young’s  modulus | Yield strength |
| P-1 | 139.34 | 1.05 | 813.24 | 3.89 | 2128.1 | 7.54 | 4038.9 | 12.35 |
| P-2 | 69.22 | 0.8 | 465.46 | 2.54 | 1270.6 | 5.68 | 2523.9 | 9.76 |
| P-4 | 3400.7 | 7.03 | 8087.7 | 15.64 | 12757 | 23.81 | 17287 | 34.58 |
| P-5 | 2946.6 | 6.09 | 6842.8 | 13.69 | 10840 | 21.68 | 15057 | 29.11 |
| Opt-P-1 | 381.98 | 1.92 | 1880.3 | 7.01 | 3967.3 | 12.86 | 6304.9 | 18.85 |
| Opt-P-2 | 156.11 | 1.06 | 917.09 | 4.34 | 2344.6 | 9.38 | 4266.8 | 15.36 |
| Opt-P-4 | 6136.8 | 10.64 | 12607 | 22.69 | 19232 | 34.62 | 25968 | 46.74 |
| Opt-P-5 | 4905.6 | 8.18 | 10274 | 17.81 | 15787 | 27.37 | 21377 | 37.05 |

**Supplementary Table 3** Numerical and experimental Young’s moduli comparison for fabricated cell models (MPa)

| Model | Numerical simulation | TPU | PA12 | SS316 | AlSi10Mg |
| --- | --- | --- | --- | --- | --- |
| P-1 | 813.24 | 0.064 | 1.83 | 320.40 | 176.00 |
| P-2 | 465.46 | 0.039 | 1.79 | 258.05 | 103.83 |
| P-4 | 8087.7 | 0.98 | 34.8 | 1403.60 | 993.67 |
| P-5 | 6842.8 | 0.69 | 31.5 | 1574.31 | 781.48 |
| Opt-P-1 | 1880.3 | 0.21 | 8.85 | 634.24 | 404.19 |
| Opt-P-2 | 917.09 | 0.085 | 3.65 | 431.42 | 212.25 |
| Opt-P-4 | 12607 | 1.65 | 59.35 | 1938.88 | 1472.81 |
| Opt-P-5 | 10274 | 1.09 | 45.8 | 2001.96 | 1350.25 |

**Supplementary Table 4** Normalized Young’s moduli comparison for fabricated cell models

| Model | Numerical simulation | TPU | PA12 | SS316 | AlSi10Mg |
| --- | --- | --- | --- | --- | --- |
| P-1 | 100.0% | 100.0% | 100.0% | 100.0% | 100.0% |
| P-2 | 57.2% | 60.9% | 97.8% | 80.5% | 59.0% |
| P-4 | 994.5% | 1531.3% | 1901.6% | 438.1% | 564.6% |
| P-5 | 841.4% | 1078.1% | 1721.3% | 491.4% | 444.0% |
| Opt-P-1 | 231.2% | 328.1% | 483.6% | 198.0% | 229.7% |
| Opt-P-2 | 112.8% | 132.8% | 199.5% | 134.7% | 120.6% |
| Opt-P-4 | 1550.2% | 2578.1% | 3243.2% | 605.1% | 836.8% |
| Opt-P-5 | 1263.3% | 1703.1% | 2502.7% | 624.8% | 767.2% |

**Supplementary Table 5** Normalized Young’s moduli comparison for fabricated cell models

| Model | Numerical simulation | TPU | PA12 |
| --- | --- | --- | --- |
| P-1 | 100.0% | 100% | 100% |
| P-2 | 57.2% | 45.9% | 51.1% |
| P-4 | 994.5% | 1152.9% | 994.3% |
| P-5 | 841.4% | 811.8% | 900.0% |
| Opt-P-1 | 231.2% | 247.1% | 252.9% |
| Opt-P-2 | 112.8% | 100.0% | 104.3% |
| Opt-P-4 | 1550.2% | 1941.2% | 1695.7% |
| Opt-P-5 | 1263.3% | 1282.4% | 1308.6% |

**Supplementary Table 6** Numerical and experimental Young’s moduli comparison for fabricated 4X4X4 array models with PA12 (MPa)

| Model (PA12) | Numerical simulation | 4X4X4-FBC | Numerical simulation | 4X4X4-PBC |
| --- | --- | --- | --- | --- |
| P-1 | 3314.69 | 13.43 | 3314.69 | 13.49 |
| Opt-P-1 | 2040.75 | 8.59 | 3154.69 | 15.18 |
| Opt-P-2 | 1349.03 | 2.36 | 2769.53 | 8.89 |
| Opt-P-4 | 12718.21 | 49.2 | 12790.53 | 51.11 |
| Opt-P-5 | 10251.31 | 39.72 | 11086.41 | 44.2 |

**Supplementary Table 7** | Numerical and experimental Young’s moduli comparison for fabricated 4X4X4 array models with AlSi10Mg (MPa)

| Model (AlSi10Mg) | Numerical simulation | 4X4X4-FBC | Numerical simulation | 4X4X4-PBC |
| --- | --- | --- | --- | --- |
| P-1 | 3314.69 | 870.73 | 3314.69 | 865 |
| Opt-P-1 | 2040.75 | 834.18 | 3154.69 | 813.21 |
| Opt-P-2 | 1349.03 | 512.16 | 2769.53 | 795.71 |
| Opt-P-4 | 12718.21 | 1730.44 | 12790.53 | 1888.77 |
| Opt-P-5 | 10251.31 | 1747.34 | 11086.41 | 1609.89 |

**Supplementary Table 8** Normalized Young’s moduli comparison for fabricated 4X4X4 models

| Model | Numerical simulation | PA12-FBC | AlSi10Mg-FBC | Numerical simulation | PA12-PBC | AlSi10Mg-PBC |
| --- | --- | --- | --- | --- | --- | --- |
| P-1 | 100.0% | 100.0% | 100.0% | 100.0% | 100.0% | 100.0% |
| Opt-P-1 | 61.6% | 64.0% | 95.8% | 95.2% | 112.5% | 94.0% |
| Opt-P-2 | 40.7% | 17.6% | 58.8% | 83.6% | 65.9% | 92.0% |
| Opt-P-4 | 383.7% | 366.3% | 198.7% | 385.9% | 378.9% | 218.4% |
| Opt-P-5 | 309.3% | 295.8% | 200.7% | 334.5% | 327.7% | 186.1% |

**Supplementary Table 9** | Elastoplastic relationship of the given material of iron

| Stress (MPa) | Plastic strain |
| --- | --- |
| 400 | 0 |
| 429.825 | 0.0114319 |
| 462.573 | 0.0290993 |
| 535.088 | 0.0748268 |
| 642.69 | 0.151732 |
| 729.24 | 0.22448 |
| 766.667 | 0.265012 |
| 797.076 | 0.302425 |
| 825.146 | 0.345035 |
| 836.842 | 0.36582 |

**Supplementary Table 10** | Material property of aluminum

| Property | Value |
| --- | --- |
| Density | 2700 kg/m^3^ |
| Young’s modulus | 70 GPa |
| Poisson’s ratio | 0.33 |
| Thermal conductivity | 238 W/(m$\cdot$K) |
| Heat capacity at constant pressure | 900 J/(Kg$\cdot$K) |

**Supplementary Table 11** | Material property of air

| Property | Value |
| --- | --- |
| Density | 1.29 kg/m^3^ |
| Speed of sound | 340 m/s |
| Relative permittivity | 1 |
| Dynamic viscosity | 17.9$\times$10^-6^ Pa$\cdot$s |
| Thermal conductivity | 0.0267 W/(m$\cdot$K) |
| Specific gas constant | 287 J/(Kg$\cdot$K) |
| Heat capacity at constant pressure | 1005 J/(Kg$\cdot$K) |

**Supplementary references**

1. S. G. Lekhnitskii, Theory of Elasticity of an Anisotropic Elastic Body, SIAM Review, 9, pp: 136-136, 1967.
2. M.A. Slawinski, Waves and Rays in Elastic Continua, World Scientific: 2nd Ed., 2010.
3. T.H.E. Kings, “Advanced mechanics of materials” 5th edition, A.P. Boresi, R.J. Schmidt and O.M. Sidebottom, Strain, 29, pp: 141-142, 1993.
4. C. Zener, Contributions to the Theory of Beta-Phase Alloys, Phys. Rev., 71, 12, pp: 846-851, 1947,
5. Z. Hashin, S. Shtrikman, A variational approach to the theory of the elastic behaviour of multiphase materials. J. Mech. Phys. Solids 11, pp: 127–140, 1963.
6. J. B. Berger, H. N. Wadley, R. M. McMeeking, Mechanical metamaterials at the theoretical limit of isotropic elastic stiffness. Nature, 543, pp: 533–537, 2017.
7. T. Tancogne-Dejean, M. Diamantopoulou, M. B. Gorji, C. Bonatti, D. Mohr, 3D plate-lattices: An emerging class of low-density metamaterial exhibiting optimal isotropic stiffness. Adv. Mater., 30, e1803334, 2018.
8. P. M. Suquet, Overall potentials and extremal surfaces of power law or ideally plastic composites. J. Mech. Phys. Solids, 41, pp: 981–1002, 1993.
9. L.J. Gibson, M.F. Ashby, Cellular Solids: Structure and Properties, Cambridge university press, 1999.
10. Y. Liu, C. Yang, P. Wei, P. Zhou, J. Du, An ODE-driven level-set density method for topology optimization. Comput. Methods Appl. Mech. Eng., 387, 114159, 2021.
11. O. Sigmund, A 99 line topology optimization code written in Matlab, Structural and Multidisciplinary Optimization, 21, pp: 120-127, 2001.
12. M.Y. Wang, X. Wang, D. Guo, A level set method for structural topology optimization, Comput. Methods Appl. Mech. Eng., 35, pp: 415-441, 2003
13. G. Allaire, F. Jouve, A.M. Toader, Structural optimization using sensitivity analysis and a level-set method, J. Comput. Phys., 194, pp: 363-393, 2004.
14. O. Al-Ketan, R.K. Abu Al-Rub, Multifunctional Mechanical Metamaterials Based on Triply Periodic Minimal Surface Lattices. Adv. Eng. Mater., 21: 1900524, 2019.
