# Supplementary material for: Ultrastiff metamaterials generated through a multilayer strategy and topology optimization
Source: Nat Commun. 2024 Apr 6;15:2984. doi: 10.1038/s41467-024-47089-8 (PMC10998847; doi:10.1038/s41467-024-47089-8)
Supplement: Supplementary file 3 — Description of Additional Supplementary Files [file 41467_2024_47089_MOESM3_ESM.pdf]

Description of Additional Supplementary Files

Supplementary Movie Legends

Supplementary Movie 1: Compressing evolution movie of the 4X4X4 model P-1

Supplementary Movie 2: Compressing evolution movie of the 4X4X4 model P-2

Supplementary Movie 3: Compressing evolution movie of the 4X4X4 model P-4

Supplementary Movie 4: Compressing evolution movie of the 4X4X4 model P-5

Supplementary Movie 5: Compressing evolution movie of the 4X4X4 model Opt-P-1

Supplementary Movie 6: Compressing evolution movie of the 4X4X4 model Opt-P-2

Supplementary Movie 7: Compressing evolution movie of the 4X4X4 model Opt-P-4

Supplementary Movie 8: Compressing evolution movie of the 4X4X4 model Opt-P-5
